# Natural allelic variation confers diversity in the regulation of flag leaf traits in wheat

**DOI:** 10.1038/s41598-024-64161-x

**Published:** 2024-06-10

**Authors:** Matías Schierenbeck, Ahmad Mohammad Alqudah, Samar Gamal Thabet, Evangelina Gabriela Avogadro, Juan Ignacio Dietz, María Rosa Simón, Andreas Börner

**Affiliations:** 1 Genebank Department, Leibniz Institute of Plant Genetics and Crop Plant Research (IPK), OT Gatersleben, Corrensstraße 3, 06466 Seeland, Germany; 2 Faculty of Agricultural Sciences and Forestry, National University of La Plata, La Plata, Argentina; 3 CONICET CCT La Plata, La Plata, Argentina; 4 Biological Science Program, Department of Biological and Environmental Sciences, College of Art and Science, Qatar University, Doha, Qatar; 5 Department of Botany, Faculty of Science, Fayoum University, Fayoum, Egypt; 6 EEA INTA Bordenave, Ruta 76 km 36, Bordenave, Argentina

**Keywords:** Flag leaf area, Bread wheat, Grain yield, GWAS, Candidate genes, FarmCPU, Plant sciences, Genetics, Agricultural genetics, Plant genetics

## Abstract

Flag leaf (FL) dimension has been reported as a key ecophysiological aspect for boosting grain yield in wheat. A worldwide winter wheat panel consisting of 261 accessions was tested to examine the phenotypical variation and identify quantitative trait nucleotides (QTNs) with candidate genes influencing FL morphology. To this end, four FL traits were evaluated during the early milk stage under two growing seasons at the Leibniz Institute of Plant Genetics and Crop Plant Research. The results showed that all leaf traits (Flag leaf length, width, area, and length/width ratio) were significantly influenced by the environments, genotypes, and environments × genotypes interactions. Then, a genome-wide association analysis was performed using 17,093 SNPs that showed 10 novel QTNs that potentially play a role in modulating FL morphology in at least two environments. Further analysis revealed 8 high-confidence candidate genes likely involved in these traits and showing high expression values from flag leaf expansion until its senescence and also during grain development. An important QTN (wsnp_RFL_Contig2177_1500201) was associated with FL width and located inside *TraesCS3B02G047300* at chromosome 3B. This gene encodes a major facilitator, sugar transporter-like, and showed the highest expression values among the candidate genes reported, suggesting their positive role in controlling flag leaf and potentially being involved in photosynthetic assimilation. Our study suggests that the detection of novel marker-trait associations and the subsequent elucidation of the genetic mechanism influencing FL morphology would be of interest for improving plant architecture, light capture, and photosynthetic efficiency during grain development.

## Introduction

The functionality and dimensions of the flag leaf (FL) play a crucial ecophysiological role in climate change adaptation and grain yield generation in cereal plants, especially in bread wheat (*Triticum aestivum* L.). Considered a staple crop for an estimated 35% of the world´s population, wheat provides 20% of the calories worldwide and is the main source of plant-based protein in human diets^1–3^. Substantial changes in agronomic processes and technological advances for crop improvement are needed in order to surpass the current annual genetic gains and meet the growing global demand for this grain and its sub-products^4^.

Flag leaf features and their architecture have been widely reported as a determining factor and crucial source for enhancing yield potential under a wide range of environments^5–7^. Flag leaf dimension is particularly important for wheat yields, due to its delayed senescence, light interception profile compared to lower strata, and its closest position to the sinks^8,9^. Photosynthesis produced by the FL in wheat contributes between 30 and 50% of the assimilates during grain development and filling^10^. Therefore, its dimension and greenness longevity correlate closely with the accumulation of dry matter in the grain^6,11,12^. The strategies are to increase the efficiency of FL area (FLA) and prolong their functionality as an essential to ensure more supply of assimilates, which in turn improve grain yield and quality^12–14^. Flag leaf length (FLL) and width (FLW) have become important traits for selection in breeding programs due to their positive correlations with grain weight, grain number per spike, and other yield-related traits^15–17^. Taking this into account, uncovering the genetic base as well as exploring genotypic variations on flag leaf architecture traits can be considered key to boosting photosynthesis efficiency, which helps increase grain yield potential^17^.

The Genome-Wide Association Study (GWAS) method, combined with novel computational approaches that enhance efficiency^18^, holds significant potential for increasing crop yield by identifying genetic variations linked to desirable traits, thereby enabling more targeted and effective breeding strategies. Recent reports studied the genetic basis and QTLs controlling FL and related morphological traits in wheat using bi-parental populations as well as diverse collections^16,17,19–22^, in addition to other cereals such as barley^23,24^ and rice^25,26^. More precisely, QTLs were underlying FLL such as *qFll-4B.1*^15^, *QFll.sicau-2D.3* and *QFll.sicau-5B.3*^16^; FLW like *QFlw.sicau-2D*^16^, *QFlw-4B*, *QFlw-5B* and *QFlw-6B*^15,27^; FLA such as *qFla-4B.1*, *qFla-5B*, *qFla-6B.2*^15^, *QFla.sicau-2D*^16^; and FLWR like *QFlr.sicau-5B*^16^ and *QFlr.cau-5A.2*^28^ were previously documented in bread wheat. Ma et al.^16^ reported that *QFlw-5B* associated with FL morphology played a pleiotropic effect on plant architecture and yield-related traits. For their part, Tu et al.^20^ analyzing seven biparental populations using Kompetitive Allele Specific PCR (KASP) reported that *QFlw.sau-SY-2D* (related to FLW) was closely located with QTLs controlling thousand kernel weight, kernel width and spikelet number per spike. Liu et al.^29^ documented that closely linked QTLs for flag leaf morphology traits such as *QFLL-4B*, *QFLW-4B*, *QFLA-4B*, and *QFLANG-4B* were found to be nearby QTLs for yield-related traits including plant height, spike length and kernel number per spike. For rice, some QTLs for spikelet number per panicle (*Gn1a*, *Gn1b*, and *SPP1*) were reported to be close to *qFLL1*, associated with FLL^26^. Promising candidate genes linked with FL morphology traits were previously documented using double haploid (DH), recombinant Inbred Lines (RILs), or F2 populations^15,21,22,30^. For instance, *TaFLW1* related to FLW was mapped on 5AL closely related to Fusarium head blight (FHB) resistance gene *Fhb5*^31^. For FLL, Muhammad et al.^10^ reported three candidate genes (*TraesCS6A01G142000*, *TraesCS5A01G533200*, and *TraesCS5A01G533300*) that revealed homology to the transcription factor basic helix-loop-helix 74 which played a role in cell elongation and plant development. These authors also reported three candidate genes located in chromosome 3A (*TraesCS3A01G452400*, *TraesCS3A01G452500,* and *TraesCS3A01G452600*) annotated as Laccase which is used for lignin polymerization and related to a wide variety of functions in plant development^32^. Despite these findings being important to understand the genetic basis of FL-related traits, these studies were performed using limited genetic resources mostly RILs and bi-parental populations, therefore, wider genetic resources such as diverse populations to discover new alleles/genes controlling such important traits is imperative.

Due to its role as the main contributor of assimilates for grain filling, FL architecture has been reported as a main ecophysiological trait for boosting grain yield potential. In the current study, a genome-wide association scan (GWAS) was implemented through the Farm-CPU algorithm to analyze 261 worldwide winter wheat accessions for FL morphology traits over 2 growing seasons. Our analysis reported 10 stable and novel quantitative trait nucleotides (QTNs) playing a role in modulating these traits. Furthermore, novel candidate genes were documented to be likely involved, showing high expression values on FL tissues through the crop cycle and interestingly, also during grain development. The identification of novel QTNs and the subsequent elucidation of the genetic mechanism related to light absorption capture and their close relation with photosynthate assimilation at the grain filling would be of interest for marker-assisted selection in wheat breeding programs.

## Materials and methods

### Plant material and field trials

A worldwide winter wheat panel consisting of 261 accessions was tested to examine the natural phenotypical variation of flag leaf-related morphology. Seeds from the whole panel were provided by the German Federal ex-situ Genebank located at the Leibniz Institute of Plant Genetics and Crop Plant Research (Gatersleben, Germany). Schierenbeck et al.^33,34^ and Supplementary Table 1 reported more information on the winter panel. The authors comply with the IUCN policy statement on research involving species at risk of extinction and the Convention on the Trade in Endangered Species of Wild Fauna and Flora. All methods were carried out in accordance with institutional, national, and international relevant guidelines and regulations.

Field trials were performed at the Leibniz Institute of Plant Genetics and Crop Plant Research during 2016–2017 and 2018–2019 following a randomized complete blocks design with three replications. Each accession was sown in 2.4 m^2^ plots (2 m long by 1.2 m wide) and contained six rows. Four flag leaf traits were measured during the early milk stage (Z73)^35^, assessing twenty random of plants per plot. FLL (flag leaf length; distance from the base to the tip of the leaf) and flag leaf width (FLW; distance from the widest part of the leaf) were measured as suggested by Liu et al.^36^, while flag leaf area (FLA = FLL × FLW × 0.75) and length/width ratio (FLWR = FLL/FLW) were calculated based on Yang et al.^19^.

### Phenotypic data analysis

GenStat Release 18 software^37,38^ was used for the analysis of variance (ANOVA) and broad-sense heritability (*H*^*2*^) as suggested by Ref.^39^. The correlation coefficient, boxplots, and variations among geographical regions were plotted using MVApp v2.0^40^.

The restricted maximum likelihood (REML) algorithm was applied for Best Linear Unbiased Estimators (BLUEs) calculations using the Linear and Nonlinear Mixed Effects Models package (nlme) in R^41^ to estimate the mean value of each accession (fixed effect) over the growing seasons (random effect).

### Genotyping and population structure

A chip of the 90 K iSELECT^42^ was used to genotype the population. After the quality check, 17,093 SNP markers were mapped according to their physical position based on IWGSC RefSeq v2.1 (http://www.wheatgenome.org/) and then used to calculate the population structure, linkage disequilibrium (LD), and GWAS scan. The PCA analysis showed that the panel clustered into three groups strongly according to their different origins: 66 genotypes (25.2%) from Central-Northern Europe mainly Germany, France, Poland, Sweden, Finland, and Great Britain; 146 genotypes (55.6%) from Eastern Europe-Western Asia like Russia, Bulgaria, Kazakhstan, Ukraine; 42 accessions (16%) from North-America mainly from USA and Canada. The remaining genotypes (3.2%) come from diverse countries worldwide (Australia, Chile, China, Japan, and India). Regarding the marker coverage, the B genome showed the highest density with 8809 SNPs (51.5%), followed by the A genome (38.6% of all markers, 6595 SNPs) and the D genome with 9.9% (1689 SNPs). The homoeologous chromosome of group 1 had the highest number of SNPs (17.96%), while the chromosomes of group 4 had only *ca.* 7%. Chromosome 5B had the highest number of SNPs with 1784 markers, while chromosome 4D had only 46 SNPs. More details of the population were published in Refs.^33,34^.

### Genome-wide association study and identifying putative candidate genes

In the current analyses, we used the FARM-CPU model through GAPIT 3 in the R environment^43^. This model was selected due to improved statistical power, efficient computing time, and prevention of model overfitting compared to other models^44^. The GWAS analyses were calculated for each environment separately and BLUE values over the environments. If the − log_10_ (p-value) of SNP passed the threshold of − log_10_ (1/number of SNP markers = 5.85e^−5^) = 4.23, the SNP was considered as a significant association QTN and used for further analysis^45,46^.

The significant QTNs that were present in the two environments were further used to identify the high-confidence (HC) putative candidate genes within an LD ± 2 Mbp interval. We used the latest version of the wheat reference genome sequence of Chinese Spring by blasting against IWGSC RefSeq annotation v2.1 (http://www.wheatgenome.org) to identify the accurate physical position of QTNs and candidate genes. Because each block of LD contains a high number of candidate genes, we have selected those that have SNPs within their physical positions. To get more molecular and cellular knowledge about these genes, the WheatMine platform was used to search for the gene ontologies (GO) and InterPro number and description (https://urgi.versailles.inra.fr/WheatMine/begin.do). The underlying genes were further examined for their association with flag leaf morphology traits using previously published literature.

Expression analysis has been done through the RNA‐Seq expression data from the Wheat Expression database (https://bar.utoronto.ca/eplant_wheat/)^47^ which includes the expression of genes for flag leaf and grain development. Gene expression is presented as TPM (Transcripts Per Kilobase Million).

## Results

### Phenotypic variations on flag leaf dimension traits

**Figure 1:**
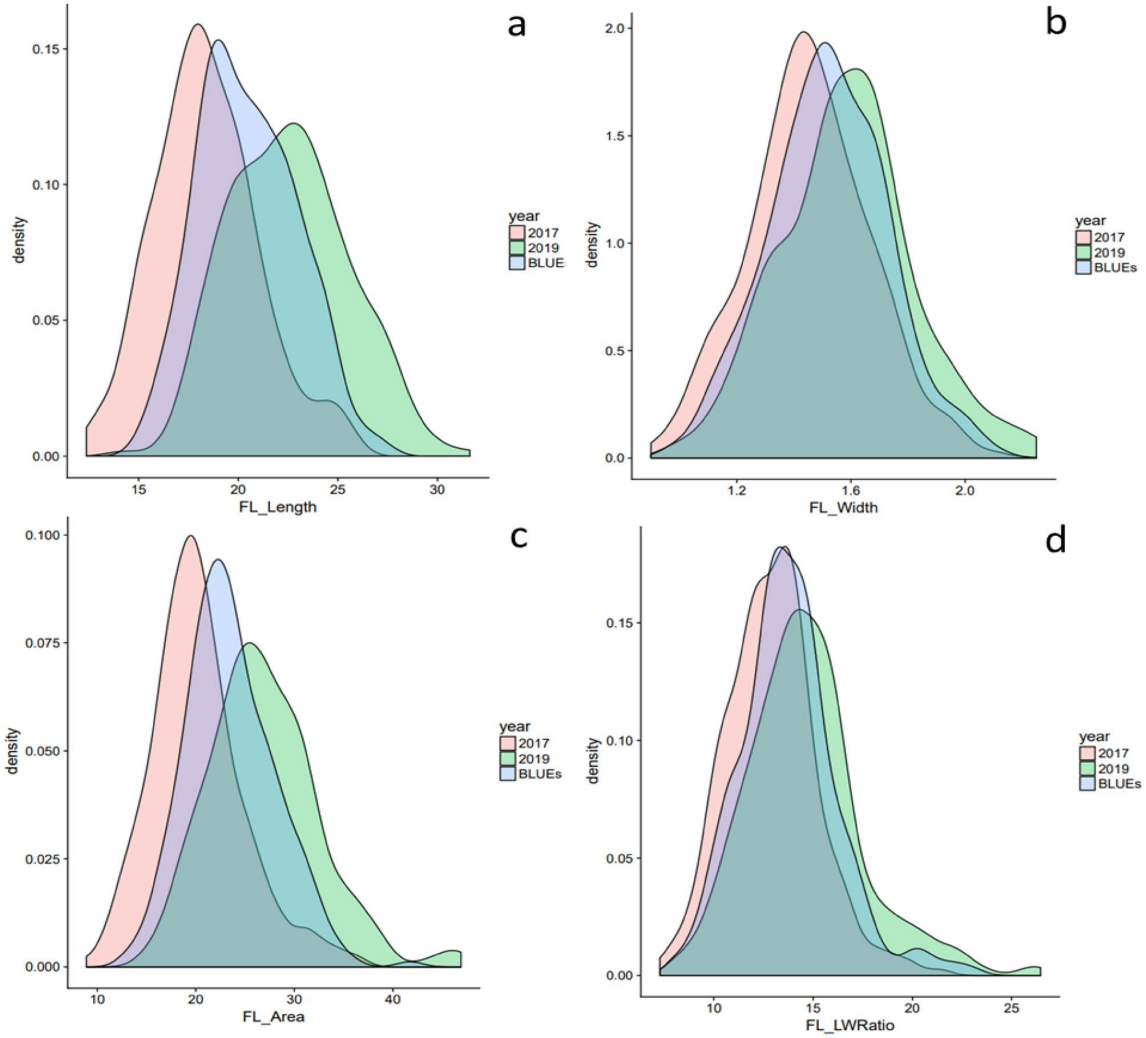
Phenotypic variation for (**a**) Flag leaf length (cm); (**b**) Flag leaf width (cm); (**c**) Flag leaf area (cm^2^) and (**d**) Flag leaf length: width ratio in 261 wheat genotypes.

**Figure 2:**
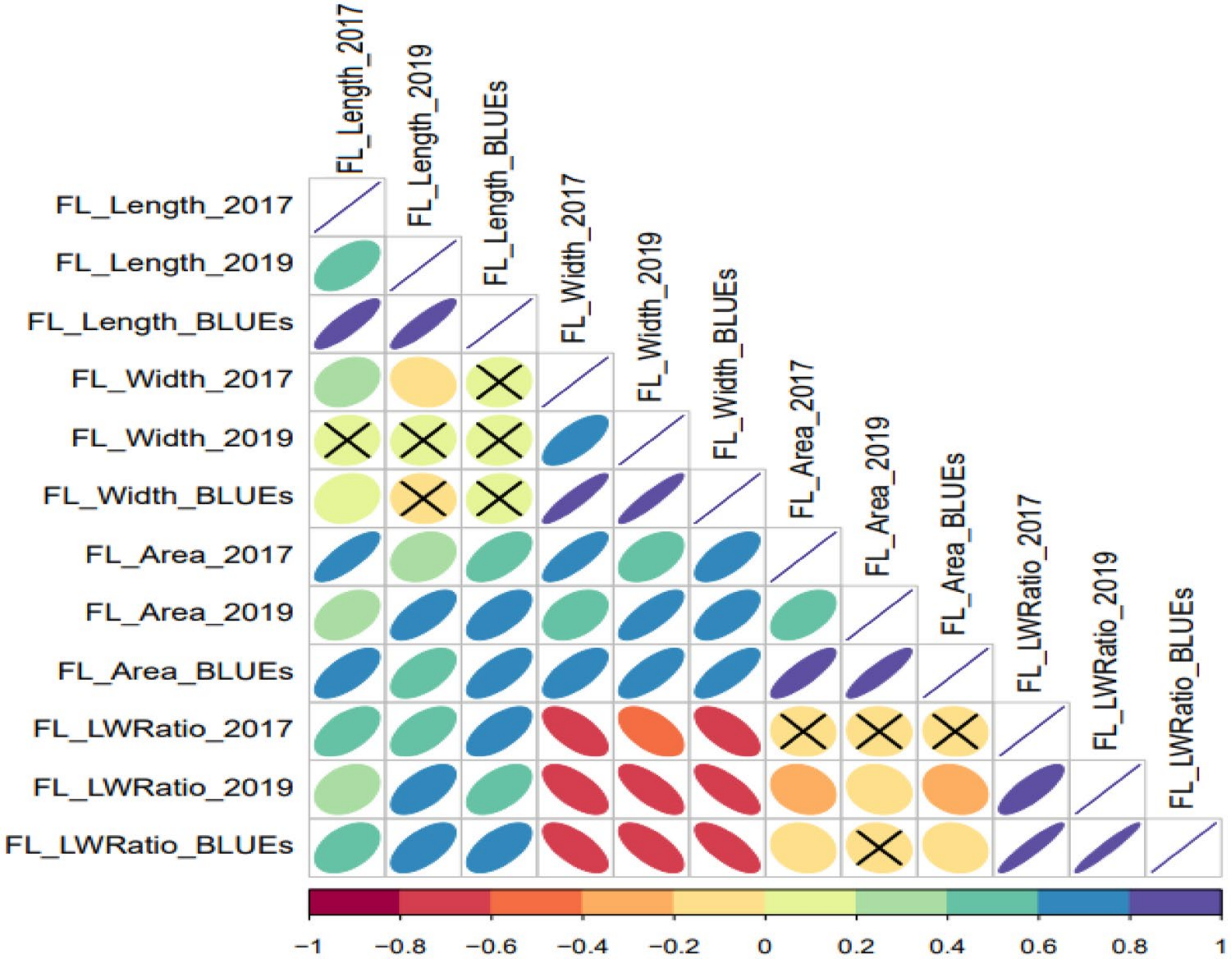
Correlation among flag leaf dimension traits in wheat genotypes. The degree of significance for all correlations across different years was P < 0.001. The color reflects the strength of the correlation. Non-significant correlations are expressed using crosses.

Flag leaf morphological traits were significantly influenced by the environments, genotypes, and *Environment* × *Genotypes* interactions (Table 1). Variations across different environments and summary statistics are shown in Table 2 and Fig. 1. Data analysis revealed extensive natural phenotypic variation with the normally distributed FL traits suggesting the suitability of the studied traits in the used association panel for additional genetic studies. A broad-sense heritability ranging from 0.72 to 0.94 was found which demonstrates that the traits are predominantly genetically controlled. Correlations among flag morphology in the different growing seasons are shown in Fig. 2. Low or non-significant correlations were reported between FLL and FLW. FLA showed a high correlation with FLL and FLW, but a slightly better adjustment with FLW (*ca.* 0.76). For their part, negative correlations were detected for FLWR and FLW, while minor negative correlations (*ca.* − 0.20) were documented for FLWR and FLA.

**Table 1:** Means square and p-value (ANOVA) of flag leaf traits in an experiment with 261 winter wheat genotypes evaluated during 2 years.

Trait	Environment (Env)	Genotype (G)	*Env* × *G*	*H*^*2*^
Flag leaf length (FLL)	9035**	48.42**	14.65**	0.72
Flag leaf width (FLW)	9.29**	0.34**	0.06**	0.93
Flag leaf area (FLA)	23,826**	159.3**	55.11**	0.75
Flag leaf length–width ratio (FLWR)	1527**	49.00**	6.18**	0.94

*H*^*2*^: broad-sense heritability.

**Significance P ≤ 0.001; *P ≤ 0.05; ns (no significant).

**Table 2:** Summary statistics of flag leaf traits in an experiment with 261 wheat genotypes evaluated during 2 years.

Trait	Env	Mean	Min	Max	Var	Median	%CV	*s.d*
Flag leaf length (FLL; cm)	2017	18.39	10.7	28	9.27	18	16.56	3.04
	2019	22.55	12.7	33.5	11.79	22.6	11.79	3.43
	BLUEs	20.48	10.7	33.5	14.7	20.2	12	3.83
Flag leaf width (FLW; cm)	2017	1.45	0.8	2.1	0.052	1.4	15.73	0.229
	2019	1.586	0.9	2.4	0.074	1.6	17.22	0.273
	BLUEs	1.52	0.8	2.4	0.068	1.5	17.14	0.261
Flag leaf area (FLA; cm^2^)	2017	20.18	6.96	40.95	44.33	19.53	25.85	5.22
	2019	26.94	12.38	50.33	44.33	26.52	24.72	6.66
	BLUEs	23.57	6.96	50.33	46.96	22.69	29.15	6.85
Flag leaf length–width ratio (FLWR)	2017	12.9	6.16	25.56	6.59	12.73	19.9	2.57
	2019	14.61	6.35	29.2	10.74	14.29	22.43	3.28
	BLUEs	13.76	6.16	29.2	9.32	13.46	17.14	3.05

Env: Environment; Min.: Minimun; Max.: Maximun; Var.: Variance; %CV: coefficient of variation; s.d: standard deviation.

**Figure 3:**
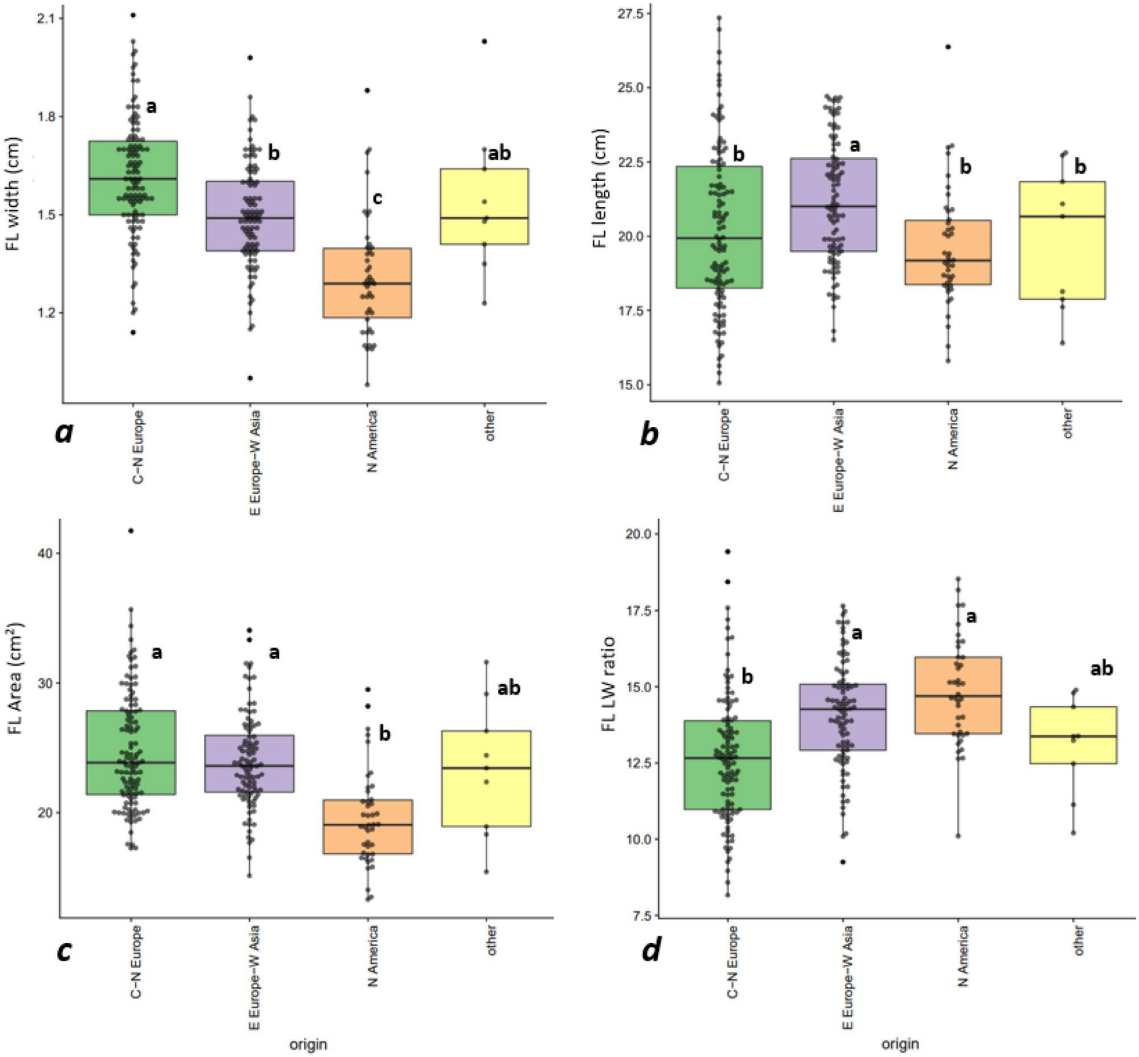
Variation on (**a**) flag leaf width; (**b**) flag leaf length; (**c**) Flag leaf area and (**d**) Flag leaf length/width ratio based on genotypes origin. C–N Europe (Central–Northern Europe; 66 genotypes); E Europe–W Asia (Eastern Europe–Western Asia; 146 genotypes); N America (North America; 42 genotypes); Other regions (7 genotypes). Matching letters are not statistically different (LSD P ≤ 0.05).

The diverse origins of the genotypes presented a differential response to FL morphology traits. Cultivars from Central and Northern Europe exhibited higher values for FLW and FLA (1.61 cm and 24.74 cm^2^, respectively) in comparision with North American cultivars (1.31 cm and 19.56 cm^2^, respectively). Genotypes from Eastern Europe-Western Asia showed intermediate values (Fig. 3).

### Genome-wide association mapping analysis

**Figure 4:**
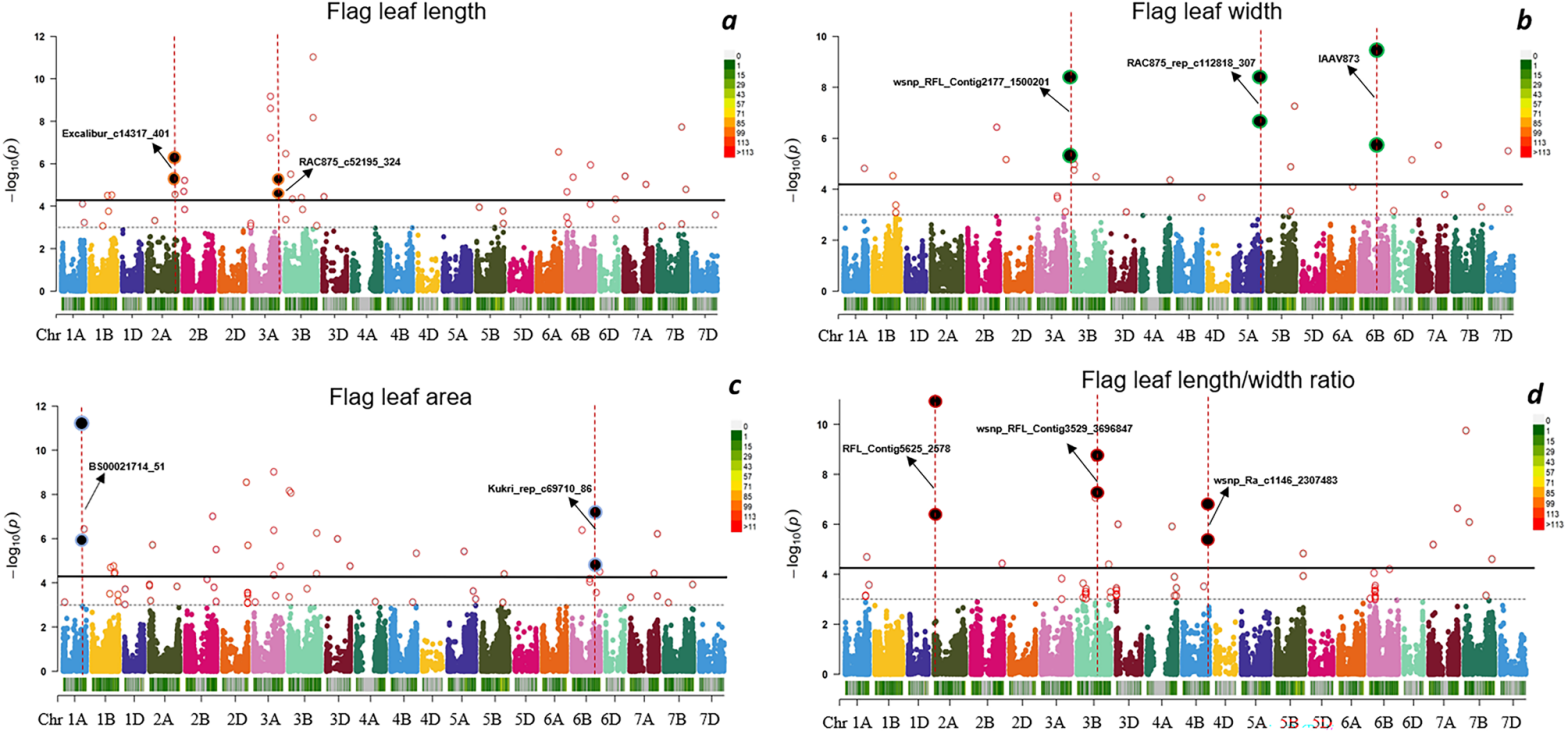
Manhattan plots showing significant marker trait associations for (**a**) Flag leaf length, (**b**) Flag leaf width, (**c**) Flag leaf area and (**d**) Flag leaf lenght/width ratio in 261 winter wheat genotypes. For each trait, dashed red lines and highlighted black circles show QTNs surpassing the significant threshold − log_10_ (5.85e^−5^) = 4.23 in two environments. Names of significant QTNs are pointed with black arrows.

Ten stable QTNs in at least two environments − log_10_ (5.85e^−5^) = 4.23 related to flag leaf morphology were detected using the FARM-CPU method. These markers were identified on chromosomes 1A (1), 2A (2), 3A (1), 3B (2), 4B (1), 5A (1) and 6B (2) (Fig. 4 and Table 3).

**Table 3:** Distribution of significant QTN (quantitative trait nucleotides) in at least two environments and candidate genes associated with flag leaf morphology traits. Significant threshold − log_10_ (5.85e^−5^) = 4.23.

Chr	Marker/synonym	Trait/effect/LOD and p-value	Marker physical (bp), genetic position (cM), and alleles	Candidate gene-Genomic location (bp)	Annotation and previous reports of candidate genes or QTNs on wheat
1A	BS00021714_51 (IWB6733)	FLArea_2017 (− 0.88 cm^2^**)** P-value: 9.70E^−7^ LOD: 6.01 FLAREA_BLUE (− 1.15 cm^2^**)** P-value: 6.72E^−12^ LOD: 11.17	490087845..490087945 78.33 cM (C–T)	*NA*	Septoria tritici blotch^48^and *Tilletia controversa* (dwarf bunt) resistance^49^
2A	RFL_Contig5625_2578 (IWB65061)	FLWR_2019 (+ 1.50) P-value: 1.17E^−11^ LOD: 10.93 FLWR_BLUE (+ 0.99) P-value: 4.57E^−7^ LOD: 6.34	13408078..13408178 25.97 cM (A–G)	*TraesCS2A02G029800* (13405669.13409022)	NB-ARC (IPR002182) Resistance to Fusarium head blight in wheat^50^ QTL on days to maturity^51^
2A	Excalibur_c14317_401 (IWB22408)	FLL_2017 (+ 0.59 cm) P-value: 5.51E^−7^ LOD: 6.29 FLL_BLUE (+ 0.45 cm) P-value: 5.72E^−6^ LOD: 5.24	780788068..780788168 119.93 cM (C–T)	*TraesCS2A02G464100* (709827740.709837331)	SIT4 phosphatase-associated protein family (IPR007587) Photosynthesis efficiency in wheat^52^
3AL	RAC875_c52195_324 (IWB58806)	FLL_2017 (0.66 cm) P-value: 6.12E^−6^ LOD:5.21 FLL_BLUE (0.53 cm) P-value: 3.12E^−5^ LOD: 4.51	711404082..711404182 (146.86 cM) A–G	*TraesCS3A02G480600* (711401772.711407423)	Farnesoic acid O-methyl transferase (IPR022041); BTB/POZ domain (IPR000210); BTB/Kelch-associated (IPR011705); Coagulation factor 5/8 C-terminal domain (IPR000421) QTLs for wheat grain quality^53^, shoot and root morphology in durum wheat^54,55^
3B	wsnp_RFL_Contig2177_1500201 (IWA8303)	FLW_2019 (+ 0.08 cm) P-value: 3.33E^−9^ LOD: 8.48 FLW_BLUE (+ 0.04 cm) P-value: 6.63E^−6^ LOD: 5.18	23959650..23959725 (37.28 cM) A–G	*TraesCS3B02G047300* (23957233.23962495)	Major facilitator, sugar transporter-like (IPR005828) QTL for leaf rust resistance hcmQTL3B.2^56^ QTL for grain yield traits MQTL3B.1^57^
3B	wsnp_RFL_Contig3529_3696847	FLWR_2019 (+ 1.30) P-value: 2.17E^−9^ − LOG_10_: 8.66 FLLWratio_BLUE (+ 1.03) P-value: 8.59E^−8^ − LOG_10_: 7.07	467095882.467095982 T–A	*TraesCS3A02G248900* (467095562.467099371)	K Homology domain, type 1 superfamily (IPR036612) QTLs for grain hardiness and quality traits^58^
4B	wsnp_Ra_c1146_2307483 (IWA7566)	FLWR_2019 (+ 0.53) P-value: 4.04E^−6^ − LOG_10_: 5.39 FLLWratio_BLUE (+ 0.53) P-value: 1.57E^−7^ − LOG_10_: 6.80	630470763..630470863 80.61 cM (T–G)	*NA*	Reduction in Grain protein content^59^ QTL affects plant height, flowering time, and days to maturity^60^
5AL	RAC875_rep_c112818_307 (IWB62176)	FLwidth_2019 (− 0.08 cm) P-value: 2.33E^−7^ LOD: 6.63 FLwidth_BLUE (− 0.08 cm) P-value: 4.01E^−9^ LOD: 8.40	613477739.613477839 (98.90 cM) A–G	*TraesCS5A02G428800* (613477485.613482016)	Longin domain (IPR010908); Synaptobrevin (IPR001388) QTLs for spike fertility and number of spikes^61,62^ QTL for leaf rust resistance^63^
6BL	IAAV873 (IWB35529)	FLwidth_2019 (0.06 cm) P-value: 1.70E^−9^ LOD: 5.77 FLwidth_BLUE (0.06 cm) P-value: 3.32E^−10^ LOD: 9.48	450644047.450644247 65.86 cM A–C	*TraesCS6B02G251400* (450643557.450653506)	MATH/TRAF domain (IPR002083)
6B	Kukri_rep_c69710_86 (IWB49928)	FLArea_2017 (+ 1.26 cm^2^**)** P-value: 6.78E− ^8^ LOD: 7.17 FLAREA_BLUE (+ 0.79 cm^2^**)** P-value: 1.97E^−5^ LOD: 4.71	618944165.618944245 71.97 cM A–G	*TraesCS6B02G353200* (618942386.618945171)	NADH:flavin oxidoreductase/NADH oxidase, N-terminal (IPR001155) Candidate genes related to chasmogamy in wheat^64^

Chr (Chromosome); cM (Centimorgan); FLL (flag leaf length); FLW (flag leaf width); FLA (flag leaf area); FLWR (flag leaf length/width ratio); LOD: logarithm of the odds; NA (no information available).

For FLL, two QTNs were identified on chromosomes 2A and 3A. These markers were *Excalibur_c14317_401* located on chromosome 2A (LOD = 5.24–6.29) increasing FLL by 0.45–0.59 cm, and *RAC875_c52195_324* on chromosome 3A (LOD = 4.51–5.21; Effect =  + 0.53/0.66 cm). The phenotypic variation explained by markers (PVE) ranged between 4.61 and 9.47% (Fig. 4; Table 3).

Three significant QTNs were detected for FLW across 3B, 5A, and 6B. The most significant markers were *IAAV873* on chromosome 6B (LOD = 5.77–9.48; Effect =  + 0.059/0.063 cm), *RAC875_rep_c112818_307* located on 5A (LOD = 6.63–8.40; Effect = − 0.08 cm) and *wsnp_RFL_Contig2177_1500201* on Chromosome 3B (LOD = 5.18–8.48; Effect =  + 0.043/0.078 cm). The PVE ranged between 4.01 and 36.18%.

For the FLA, a total of two QTNs were reported on chromosomes 1A and 6B. *BS00021714_51* located in 1A (LOD = 6.01–11.17) reduced FLA by − 0.88 to 1.15 cm^2^) while *Kukri_rep_c69710_86* in 6B (LOD = 4.71–7.17) showed a positive effect on FLA ranging + 0.79 to 1.26 cm^2^. The PVE by markers fluctuated between 3.66 and 17.18%.

For the FLWR, three QTNs were detected on chromosomes 2A, 3B, and 4B. Most significant ones were *RFL_Contig5625_2578* on chromosome 2A (LOD = 6.34–10.93; Effect =  + 0.99/1.50); *wsnp_RFL_Contig3529_3696847* in chromosome 3B (LOD = 7.06–8.66; Effect =  + 1.03/1.30) and *wsnp_Ra_c1146_2307483* located on 4B (LOD = 5.39–6.80; Effect =  + 0.52/0.53). The PVE ranged between 5.65 and 32.49% (Fig. 4; Table 3).

### Candidate genes underlying flag leaf morphology traits in winter wheat

After detecting stable QTNs a further analysis exhibited eight novel high-confidence candidate genes influencing flag leaf morphological traits on chromosomes 2A (2), 3A, 3B (2), 5A and 6B (2) (Table 3). Based on GWAS and LD outputs, we mined the most reliable candidate genes affecting FL dimensions (Supplementary Figs. S1 to S6).

For example, on chromosome 3A a strong and significant QTN (*RAC875_c52195_324*) was detected and located inside exon 2 of the *TraesCS3A02G480600* gene at position 711401772–711407423 bp (Supplementary Fig. S1a, Table 3). The candidate gene encodes the BTB/POZ domain (IPR000210) which harbors the phenotypic variation of FLL (Supplementary Fig. S1b). For this QTN, allelic effects showed longer flag leaf length for genotypes carrying the A allele compared to G, demonstrating that those varieties would be used as elites for marker-assisted selection (Supplementary Fig. S1c).

Interestingly, our study identified a highly significant SNP, namely *wsnp_RFL_Contig2177_1500201* at chromosome 3B (Supplementary Fig. S2a). This SNP located on exon 6 of gene *TraesCS3B02G047300* at position 23957233–23962495 bp, encodes a Major facilitator, sugar transporter-like (IPR005828), which harbors variations on FLW (Supplementary Fig. S2b). Moreover, the allelic effect for this QTN showed longer FLW for genotypes carrying the G allele, demonstrating that those genotypes would accumulate more sugars via the photosynthesis process that in turn may lead to increases in kernel weight (Supplementary Fig. S2c).

Another significant SNP, namely *RAC875_rep_c112818_307* was located on chromosome 5A (Supplementary Fig. S3a). This SNP located inside exon 4 of the *TraesCS5A02G428800* gene at position 613477485–613482016 bp, encodes Synaptobrevin that also harbors variations on FLW (Supplementary Fig. S3b). Further, accessions carrying the G allele showed a positive effect on flag leaf width compared to accessions carrying the A allele (Supplementary Fig. S3c).

The QTN *IAAV873* was located on chromosome 6B and was associated with FLW. This QTN was located within exon 1 of *TraesCS6B02G251400* at position 450644047–450644247 bp which encodes the MATH/TRAF domain (Supplementary Fig. S4a,b). The TRAF domain has a novel positive contributor to plant immunity that could improve plant growth and development via regulating photosynthetic assimilation in flag leaf. Accessions carrying the C allele showed a positive effect on flag leaf width compared to accessions carrying the A allele (Supplementary Fig. S4c), suggesting that those alleles could be used in marker-assisted selection by wheat breeding programs. Another potential candidate genes with their functions and previous reports were indicated in Table 3 and the supplementary figures (Figs. S5 and Fig. S6). Our results would indicate the potential of flag leaf trait enhancement to improve radiation capture and photosynthetic efficiency during grain filling.

**Figure 5:**
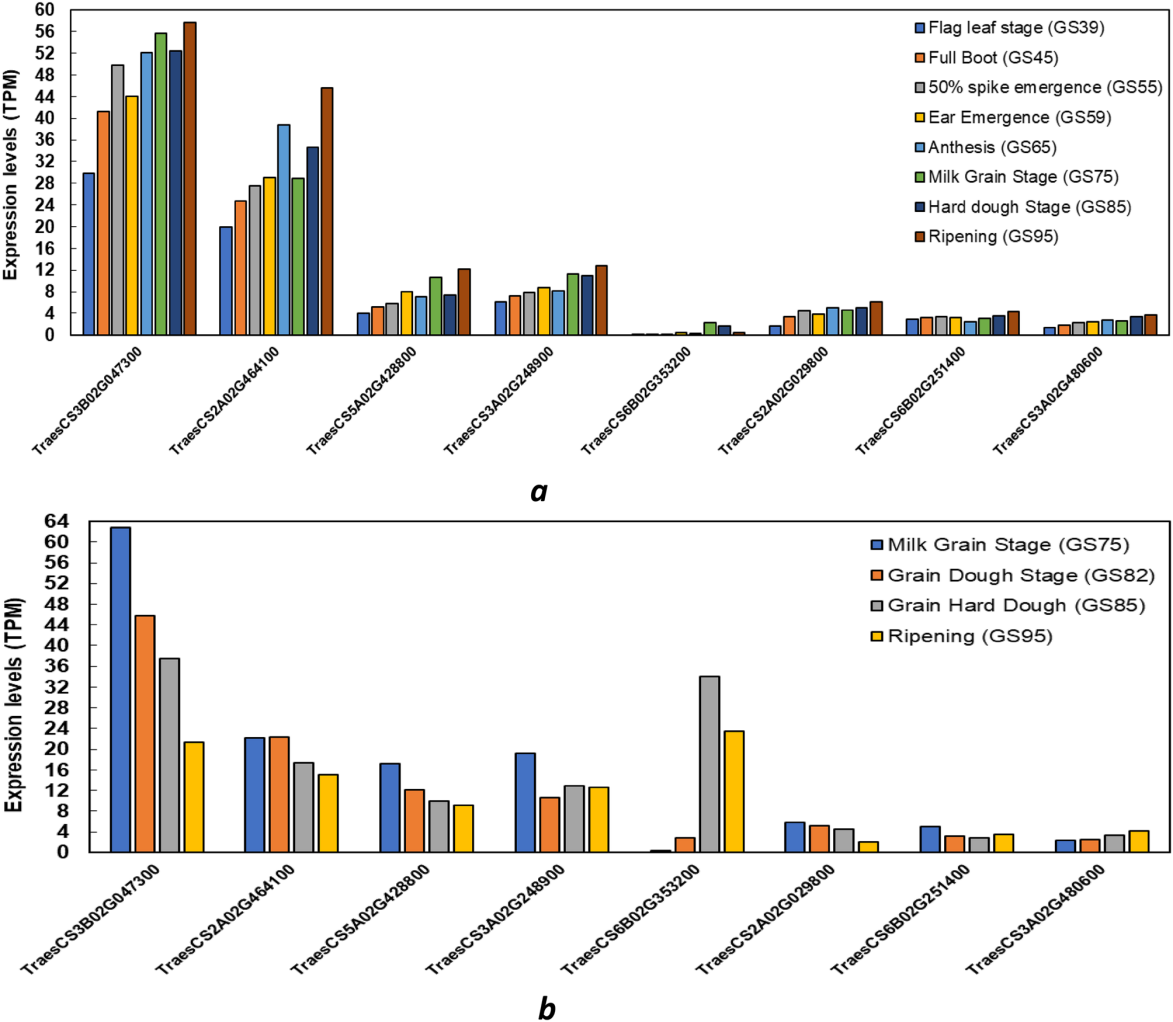
Expression value TPM (Transcripts Per Kilobase Million) of candidate genes in (**a**) flag leaf from flag leaf stage (GS39) to ripening (GS95) and (**b**) grain development (GS75 to GS95).

The expression analysis of the eight high-confidence candidate genes showed a wide range of gene expression (Fig. 5). High expression values in flag leaves and interestingly also in grain development in different developmental stages were reported for *TraesCS3B02G047300*, *TraesCS2A02G464100*, *TraesCS5A02G428800*, and *TraesCS3A02G248900* while intermediate values were detected for *TraesCS6B02G353200*, *TraesCS2A02G029800*, *TraesCS6B02G251400*, and* TraesCS3A02G480600.*

## Discussion

### Phenotypic variation

Exploring genotypic variations and uncovering the genetic basis of flag leaf architecture traits have been considered key traits for increasing photosynthesis efficiency and grain yield potential^17^. The current study revealed extensive phenotypic variation in flag leaf traits across the 261 winter wheat accessions, with high heritability reported for all traits. Furthermore, a high correlation among environments was observed, suggesting that these traits are predominantly genetically controlled. Coinciding with Ref.^27^, increasing FLW is an effective approach for FLA enhancement compared to FLL. Previous reports using association panels and biparental populations also documented high heritabilities and positive correlations among flag leaf morphological traits and grain yield parameters^21^ suggesting FL size optimization as an appropriate breeding approach for increasing wheat yield potential^20^.

The origins of the accessions used in the study were documented to exhibit a diverse range of flag leaf morphology traits, thereby demonstrating the diversity of such traits. Cultivars from Central and Northern Europe showed higher values for FLW and FLA compared to North American and Eastern Europe-Western Asia accessions. Extensive phenotypic variation among these traits has been widely reported mainly in DH and RIL populations^16,22^ and diverse panels^17,30^, however, an association on the genotype´s origin as described here has not been previously documented. Future studies should assess if differences in FL dimensions are related to an indirect selection effect concerning the photosynthetic active radiation available at different latitudes.

### Novel QTNs and Candidate genes linked with flag leaf morphology

We reported 10 novel and stable QTNs (LOD ≥ 4.23) in at least two environments related to flag leaf dimensions over seven chromosomes of the wheat genome (1A, 2A, 3A, 3B, 4B, 5A, 5B, and 6B). As previously reported, flag leaf morphological traits are quantitative traits controlled by multiple genes^6,65^. Recently some authors have compiled previous reports related to QTL involved in flag morphological traits on different wheat chromosomes^22,65^. Even though previous efforts mainly using RILs and DH population have documented markers linked with flag leaf morphology on 1A^22^, 1B^19,36^, 2A,^19^, 2B^19^, 3A^36^, 3B^22^, 4A^22^, 4B^22^, 5A^19,36^, 5B^19^, 6B^36^, 7A^22^, 7B and 7D^36^, those QTNs documented in this work have not been previously reported. These findings would indicate the potential of these novel QTNs for improving flag leaf architecture and therefore, boosting light capture and photosynthetic efficiency during grain filling.

In the same sense, a further bioinformatics analysis revealed eight novel candidate genes influencing flag leaf morphology on chromosomes 2A (2), 3A, 3B (2), 5A, and 6B (2). Although these candidate genes have been documented for diverse traits in wheat (such as disease resistance, grain quality traits, grain yield, plant growth and development) none of them have been reported to be associated with traits related to flag leaf morphology in wheat, which highlights the importance of our work (Table 3).

A significant QTN (*RAC875_c52195_324*) was detected on chromosome 3A and located inside the *TraesCS3A02G480600* gene at position 711401772–711407423 bp. This gene encodes the BTB/POZ domain (IPR000210) which controls the phenotypic variation of FL length. Our results revealed that the allelic variation for marker detected that the accessions carrying A allele (229 genotypes) presented longer flag leaves compared to genotypes carrying the G allele (32 genotypes) (Supplementary Fig. S1). A recent study by^22^ detected 2262 putative genes related to flag leaf size within the MQTL regions which mainly encode the F-box-like domain proteins, protein kinases, and BTB/POZ domain-containing proteins, suggesting their crucial roles in regulating leaf growth and development in *Arabidopsis*. Shariatipour et al.^66^ also reported eight wheat homologs for rice genes located on the *MQTL-4B.5*, *MQTL-5B.3*, *MQTL-7A.1*, *MQTL-7B.1*, and *MQTL-7D.2*, respectively, demonstrating the implication of these homolog genes in various biological processes associated with leaf size and chlorophyll content in rice and suggesting their involvement in the regulation of leaf size in wheat. Moreover, the effect of *RAC875_c52195_324* on wheat grain quality traits has been also reported^53^, the response that would be associated with the expression levels here reported for the candidate genes during grain filling (Table 3; Fig. 5b).

Interestingly, our study identified a highly significant SNP, namely *wsnp_RFL_Contig2177_1500201* on chromosome 3B.This QTN has also been reported to play a role in leaf rust resistance (*hcmQTL3B.2*)^56^ and grain yield-related traits (*MQTL3B.1*)^57^ (Table 3). This SNP is annotated as the candidate gene *TraesCS3B02G047300* and encodes a major facilitator, sugar transporter-like (IPR005828) that harbors the variation of the FLW. The allelic variation for this marker showed that cultivars with the G allele (33) showed wider flag leaves compared to those carrying the A allele (228 genotypes) (Supplementary Fig. S2). The sugar transporter proteins (STPs) play important roles in plant growth development, signal transmission, and cellular ion homeostasis under certain biotic and abiotic stress tolerance^67^. To date, it has been established that sugars are produced by photosynthesis and distributed mainly as sucrose through the phloem to other parts of the plant. Some of the sucrose is unloaded directly into the sink organs via the symplast whereas other sucrose is carried over long distances to the sink organs. Sucrose invertase breaks down sucrose into glucose and fructose, which produces apoplastic sugar, which is then absorbed through transmembrane absorption and transported to the sink cells by sugar transporters (STP)^68^. Since the distribution of sugars between assimilate-exporting source tissues and sugar-consuming sink tissues is crucial for plant growth and development, sugar transport in plants is considered an important research topic with economic significance for a food-secure world. This response could explain the higher expression values among all the candidates genes reported in flag leaf tissues and grain development reported here for this candidate gene (Fig. 5a,b). In *Arabidopsis,* more than 50 MSTs were identified and the STP subfamily is comprised of 14 monosaccharide/H^+^ symporters^69^. The STP subfamily encodes H^+^-importing monosaccharide transporters, which can transfer diverse hexoses and/or pentoses but not sucrose^70^. One of the main roles of STPs, which are almost all high-affinity hexose transporters with specialized expression in tissues, is to increase the sink needed for photosynthate redistribution^69^. Huai et al.^71^ reported that the ABA-induced sugar transporter *TaSTP6* increases sugar supply and promotes fungal infection in wheat. In *Arabidopsis*, the expression of *STP13* is greatly increased when challenged with *Botrytis cinerea*^72^. In maize, expression of Sucrose Transporter 1 (*ZmSUT1*) is enhanced in response to the biotrophic and necrotrophic development of the pathogen *Colletotrichum graminícola*^73^. Taken together, these processes are essential for preserving source/sink characteristics and hormonal signals, which play an important role in the whole plant development, cell growth, especially leaf-related traits, and osmotic homeostasis under certain biotic and abiotic challenges.

Another significant SNP, namely *RAC875_rep_c112818_307*, was located on chromosome 5A and has been previously related to spike fertility^61^, number of spikes^62^ and leaf rust resistance^63^. This SNP, annotated as the candidate *TraesCS5A02G428800* at position 613477485–613482016 bp, encodes Synaptobrevin which harbors the variation of the flag leaf width. We reported that genotypes carrying G allele (240 genotypes) presented wider flag leaves compared to those with A allele (21 genotypes) (Supplementary Fig. S3). Wang et al.^74^ identified *TaSYP137*, an R-SNARE subfamily gene, and *TaVAMP723*, from wheat as having long synaptobrevin domains. An evolutionary analysis of the genes *TaSYP137* and *TaVAMP723* shows that they are closely genetically related to XP_037417660.1 and XP_037439902.1, with high degrees of similarity to homologous proteins in other species. This suggests that the TaSYP137/TaVAMP723 protein may perform similar biological functions in plant development and response to biotic and abiotic stresses, similar to the corresponding proteins in other species.

Ultimately, the QTN *IAAV873* is located on chromosome 6B and was associated with flag leaf width and annotated as *TraesCS6B02G251400* at position 450644047–450644247 bp which encodes the MATH/TRAF domain. For this marker, genotypes carrying A allele (151 genotypes) presented wider flag leaves compared to those carrying the C allele (110 genotypes) (Supplementary Fig. S4). The tumor necrosis factor receptor (TNF-R)-associated factor (TRAF) domain, also referred to as the meprin and TRAF-C homology (MATH) domain, is a protein–protein interaction structure that can be found in diverse species^75^. Immune receptors play important roles in the perception of pathogens and the initiation of immune responses in both plants and animals. A study by Ao et al.^76^ identified mutations in *TRAF CANDIDATE 1b* (*TC1b*), a gene encoding a protein with four tumor necrosis factor receptor-associated factor (TRAF) domains that were shown to suppress *snc1* phenotypes underlying immune receptors. TC1b also does not physically associate with SNC1, affect SNC1 accumulation, or affect signaling of the downstream helper NLRs represented by ACTIVATED DISEASE RESISTANCE PROTEIN 1-L2 (ADR1-L2), suggesting that TC1b uniquely impacts snc1 autoimmunity. Overall, uncovering the TRAF domain protein TC1b as a novel positive contributor to plant immunity is of high importance for improving plant growth and development via regulating photosynthetic assimilation in flag leaf.

This study provides desirable alleles for FL optimization and is useful for wheat breeding strategies. In this sense, we further described that the allelic variation within the CG associated with flag leaf morphology affects several cellular and metabolic processes, leading to effects on leaf growth and senescence regulation, chlorophyll content, and prolongation of the grain-filling period. Future studies will seek to expand the set of genotypes and to utilise novel multilocus GWAS models with the objective of reducing the computation time and increasing the accuracy of QTN detection^18^. Moreover, the detection of novel QTNs and the subsequent elucidation of the genetic mechanism influencing flag leaf dimensions would be of interest for improving plant architecture, radiation capture, and photosynthetic efficiency during grain filling.

## Conclusions

Due to its role as the main contributor of assimilates for grain filling, flag leaf architecture has been reported as a main ecophysiological trait for boosting grain yield potential. High natural variation in leaf traits plays a vital role in improving grain yield and environmental stress adaptation. Using a worldwide winter wheat panel, ten stable and novel QTNs were detected playing a role in controlling flag leaf morphology. Furthermore, new candidate genes were reported to be likely involved in these traits, showing high expression values in flag leaf tissues through the crop cycle and interestingly, also during grain development. The obtention of novel QTNs linked and the subsequent elucidation of the genetic mechanism related to light absorption capture and their close relation with photosynthate assimilation at the grain filling would be of interest for marker-assisted selection in wheat breeding programs.

### Supplementary Information

Supplementary Figures.

Supplementary Table 1.

## Data Availability

The datasets generated during and/or analyzed during the current study are available from the corresponding author on reasonable request.

## Abbreviations

FL: Flag leaf
FLL: Flag leaf length
FLW: Flag leaf width
FLA: Flag leaf area
FLWR: Flag leaf length/width ratio
QTN: Quantitative trait nucleotide
QTL: Quantitative trait loci
SNP: Single nucleotide polymorphism
