# Supplementary material for: Natural allelic variation confers diversity in the regulation of flag leaf traits in wheat
Source: Sci Rep. 2024 Jun 10;14:13316. doi: 10.1038/s41598-024-64161-x (PMC11164900; doi:10.1038/s41598-024-64161-x)

## Slide 1
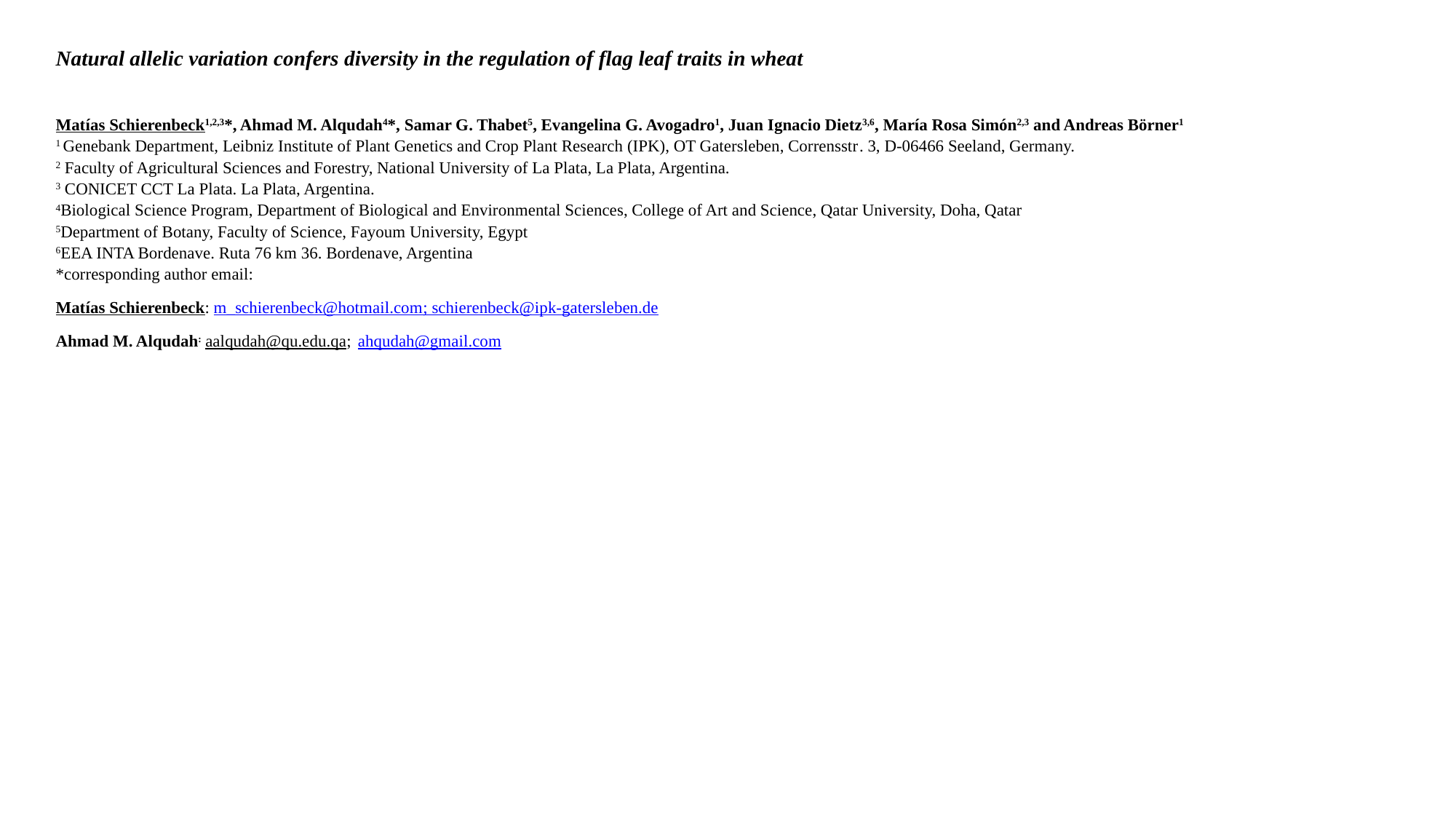

Natural allelic variation confers diversity in the regulation of flag leaf traits in wheat
Matías Schierenbeck1,2,3*, Ahmad M. Alqudah4*, Samar G. Thabet5, Evangelina G. Avogadro1, Juan Ignacio Dietz3,6, María Rosa Simón2,3 and Andreas Börner1
1 Genebank Department, Leibniz Institute of Plant Genetics and Crop Plant Research (IPK), OT Gatersleben, Corrensstr. 3, D-06466 Seeland, Germany.
2 Faculty of Agricultural Sciences and Forestry, National University of La Plata, La Plata, Argentina.
3 CONICET CCT La Plata. La Plata, Argentina.
4Biological Science Program, Department of Biological and Environmental Sciences, College of Art and Science, Qatar University, Doha, Qatar
5Department of Botany, Faculty of Science, Fayoum University, Egypt
6EEA INTA Bordenave. Ruta 76 km 36. Bordenave, Argentina
*corresponding author email:
Matías Schierenbeck: m_schierenbeck@hotmail.com; schierenbeck@ipk-gatersleben.de
Ahmad M. Alqudah: aalqudah@qu.edu.qa; ahqudah@gmail.com

## Slide 2
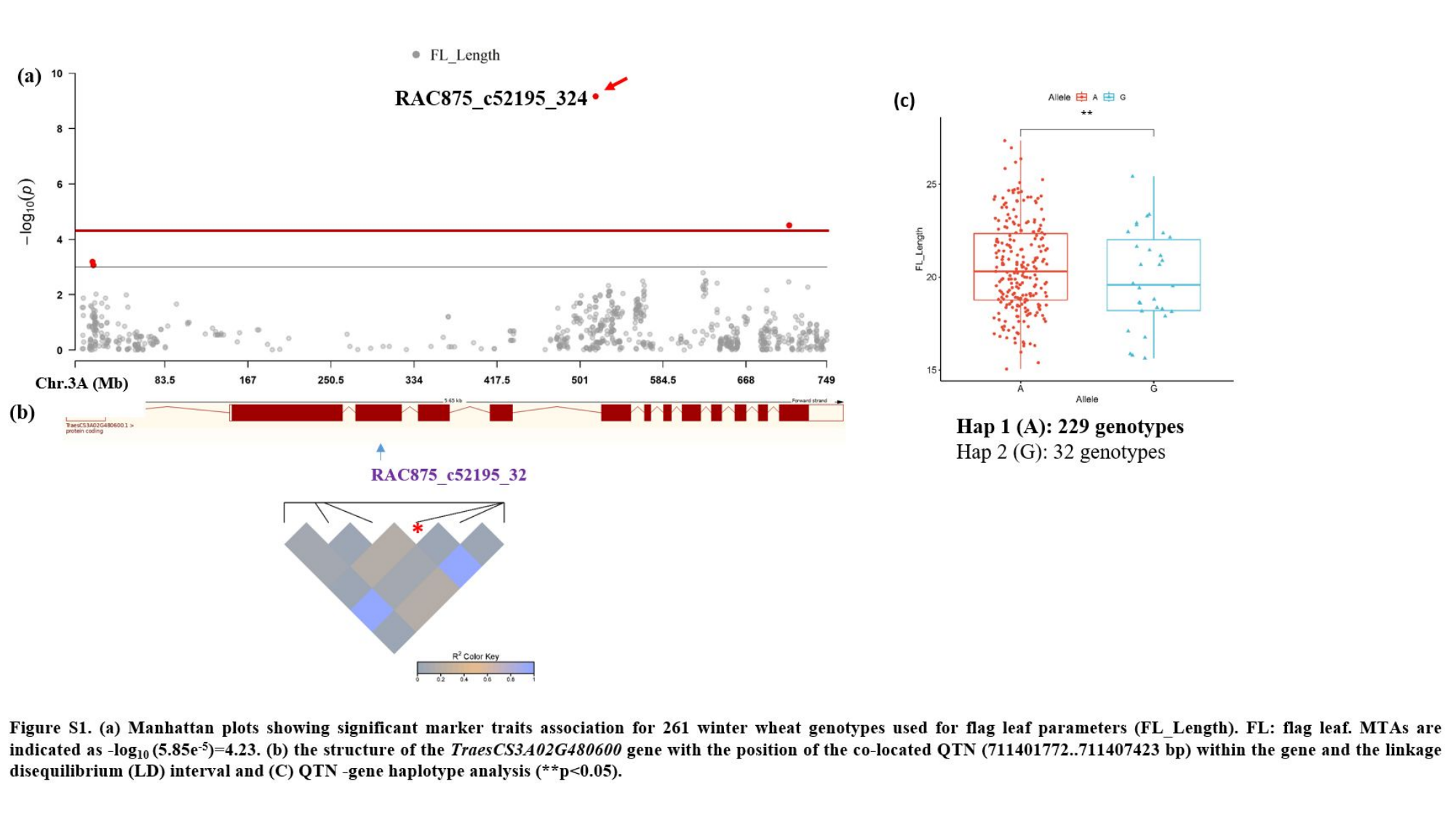

## Slide 3
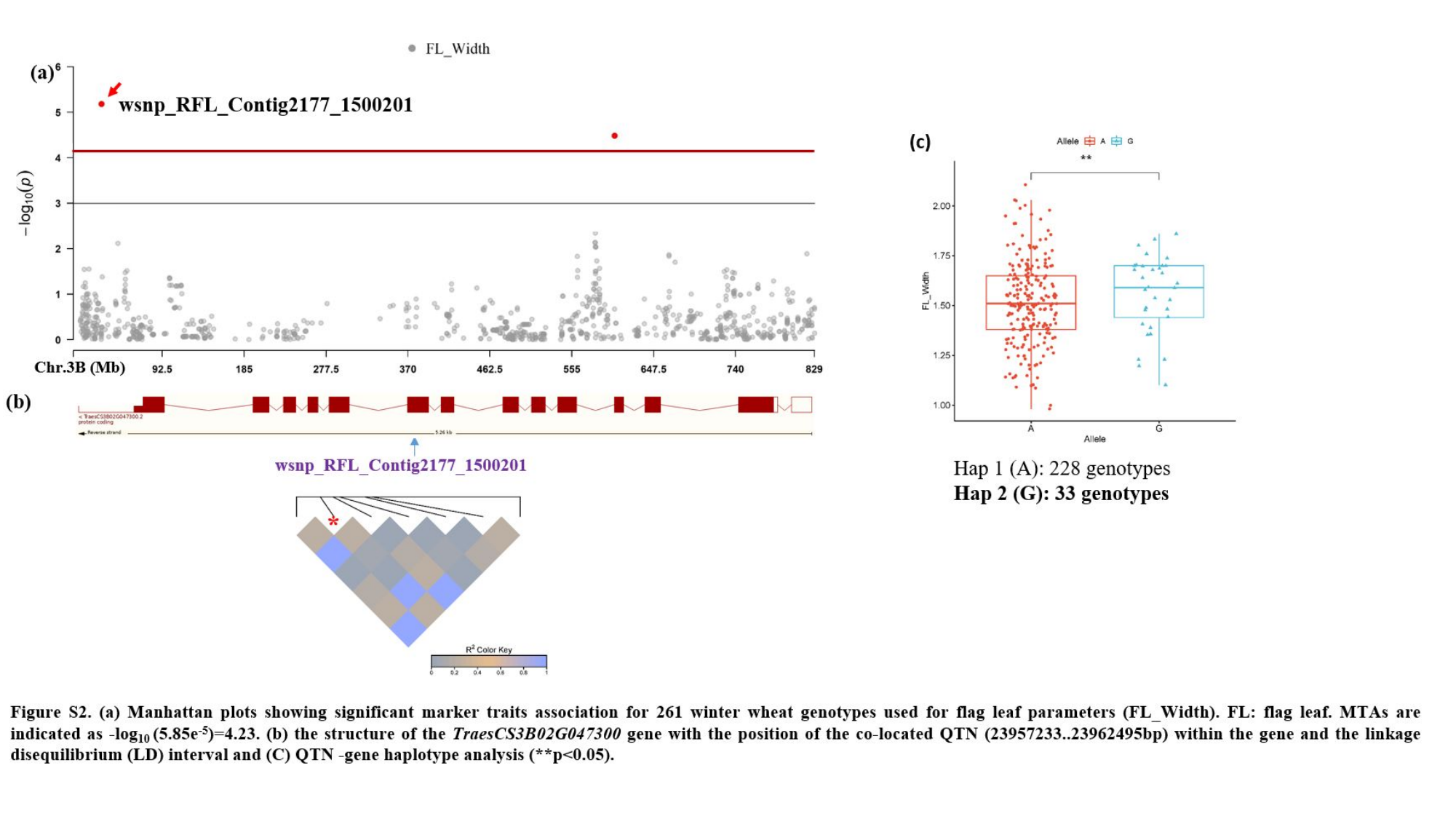

## Slide 4
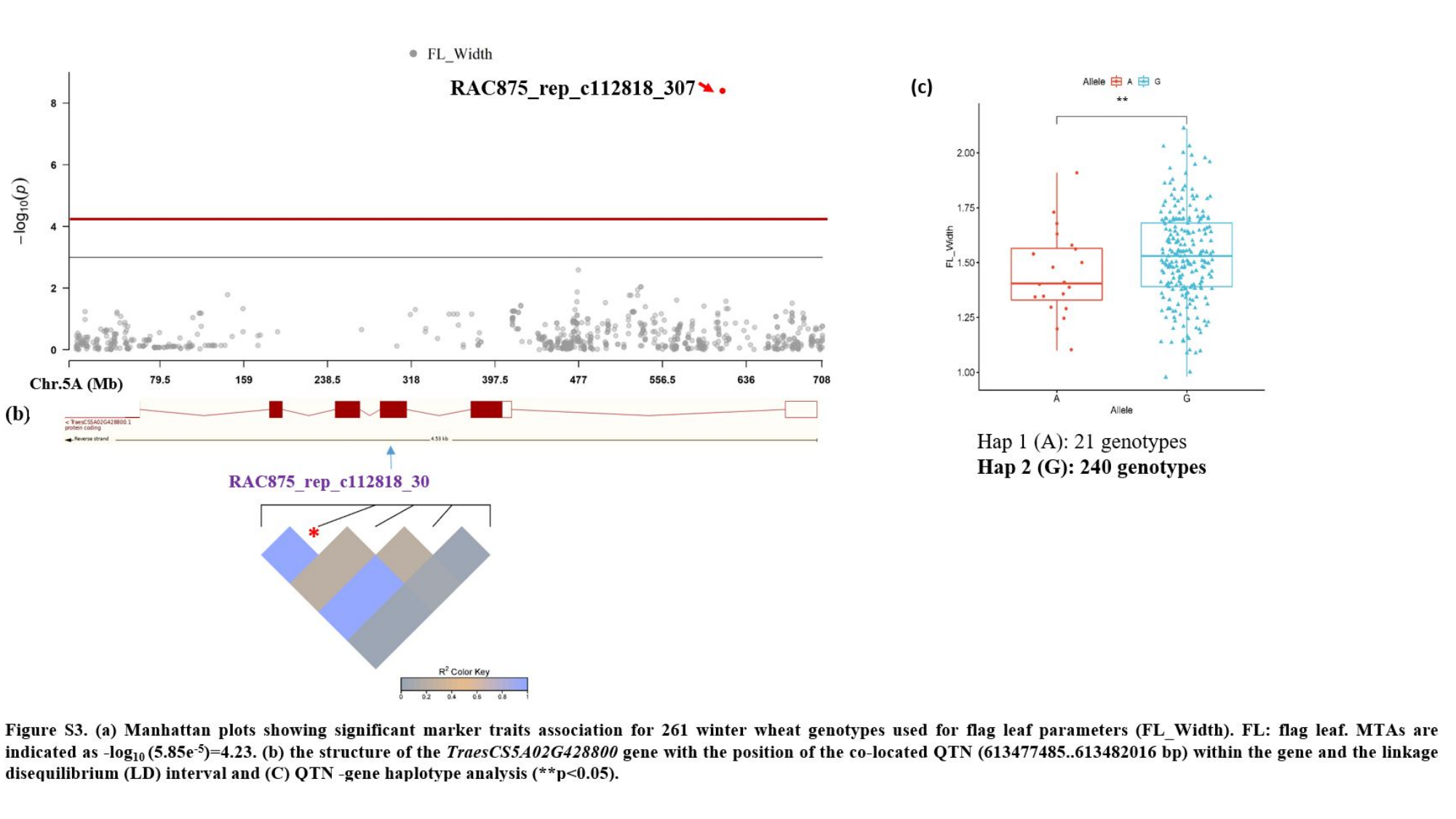

## Slide 5
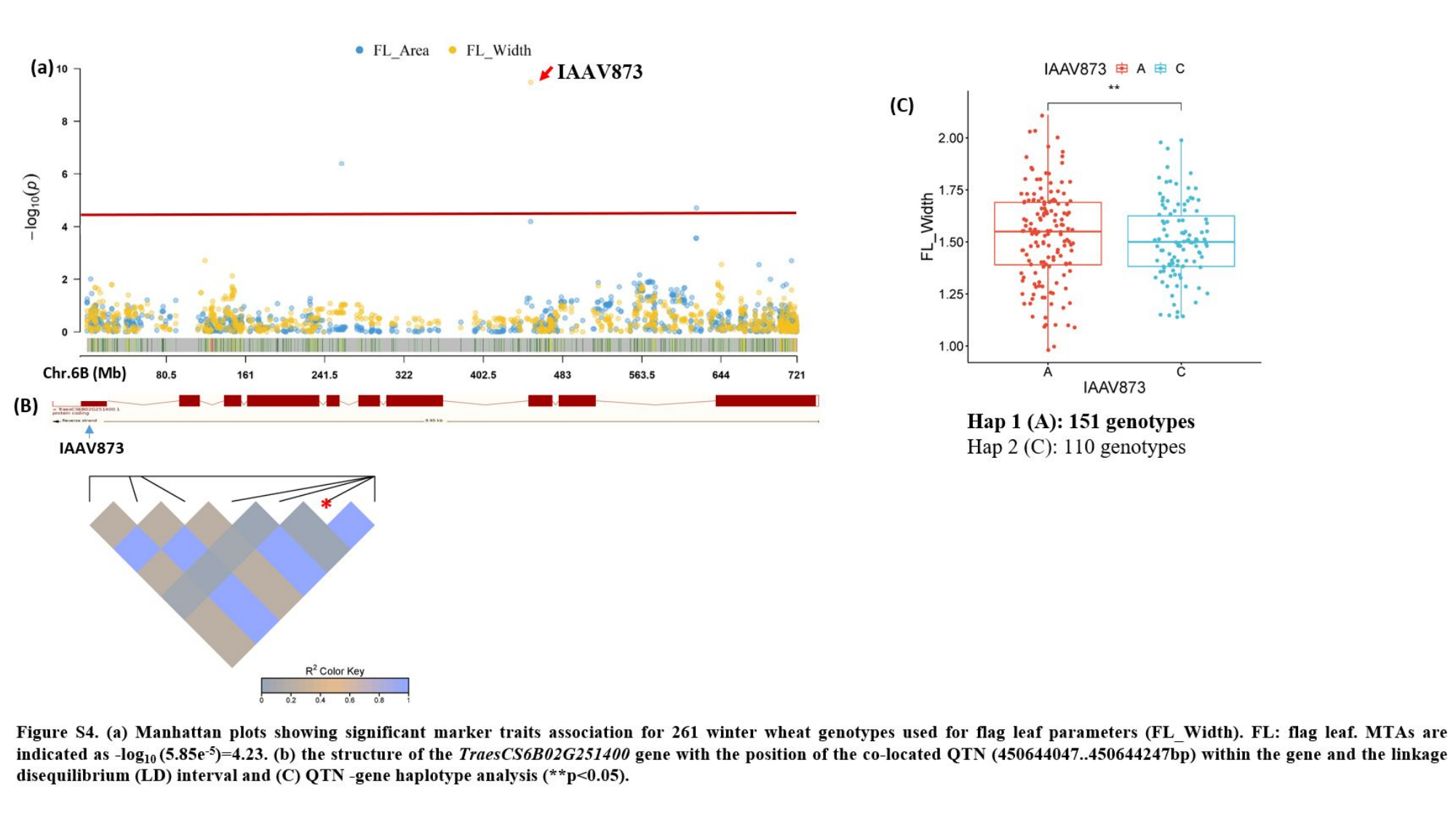

## Slide 6
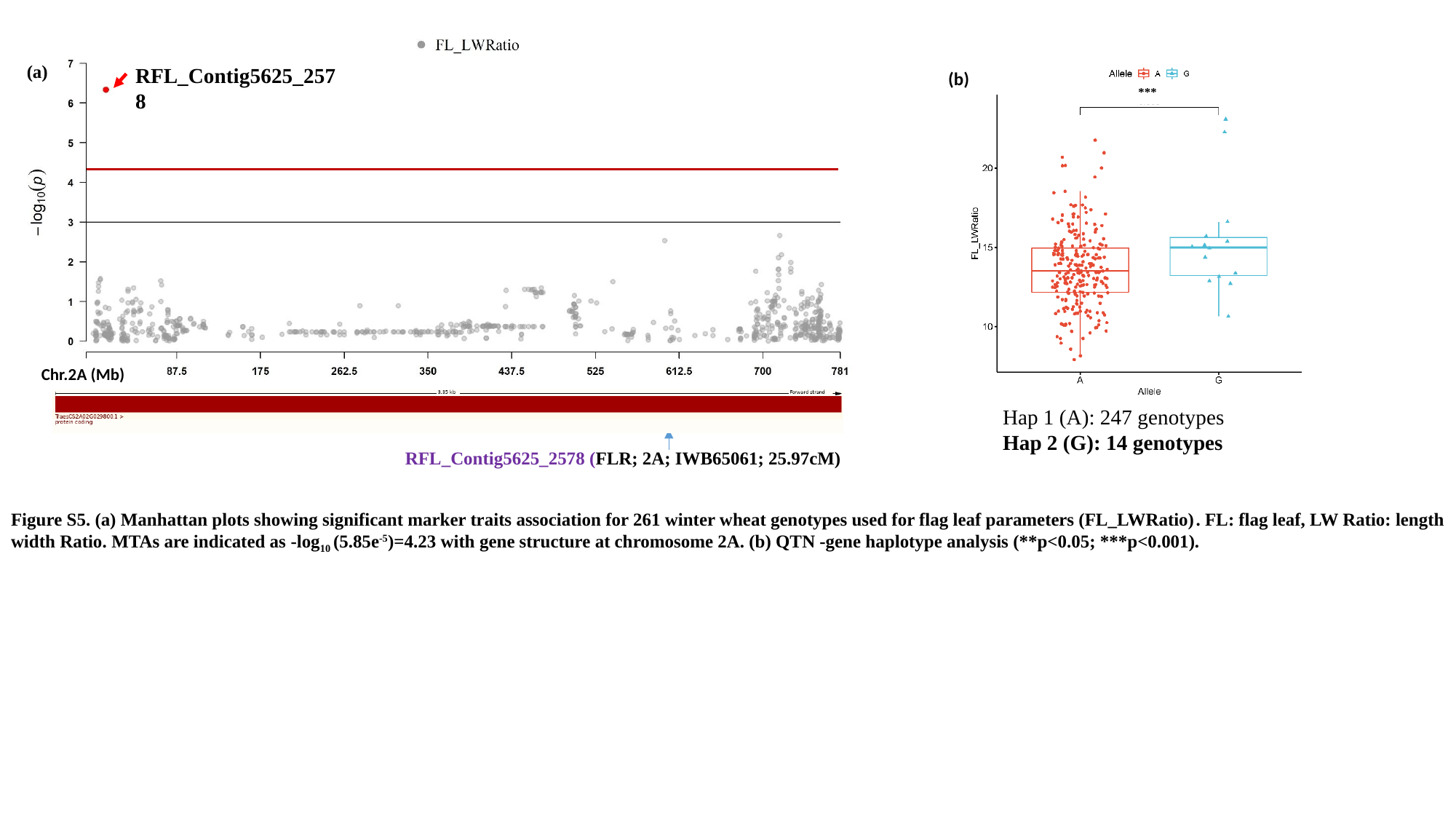

RFL_Contig5625_2578
(a)
(b)
***
Hap 1 (A): 247 genotypes
Hap 2 (G): 14 genotypes
Chr.2A (Mb)
RFL_Contig5625_2578 (FLR; 2A; IWB65061; 25.97cM)
Figure S5. (a) Manhattan plots showing significant marker traits association for 261 winter wheat genotypes used for flag leaf parameters (FL_LWRatio). FL: flag leaf, LW Ratio: length width Ratio. MTAs are indicated as -log10 (5.85e-5)=4.23 with gene structure at chromosome 2A. (b) QTN -gene haplotype analysis (**p<0.05; ***p<0.001).

## Slide 7
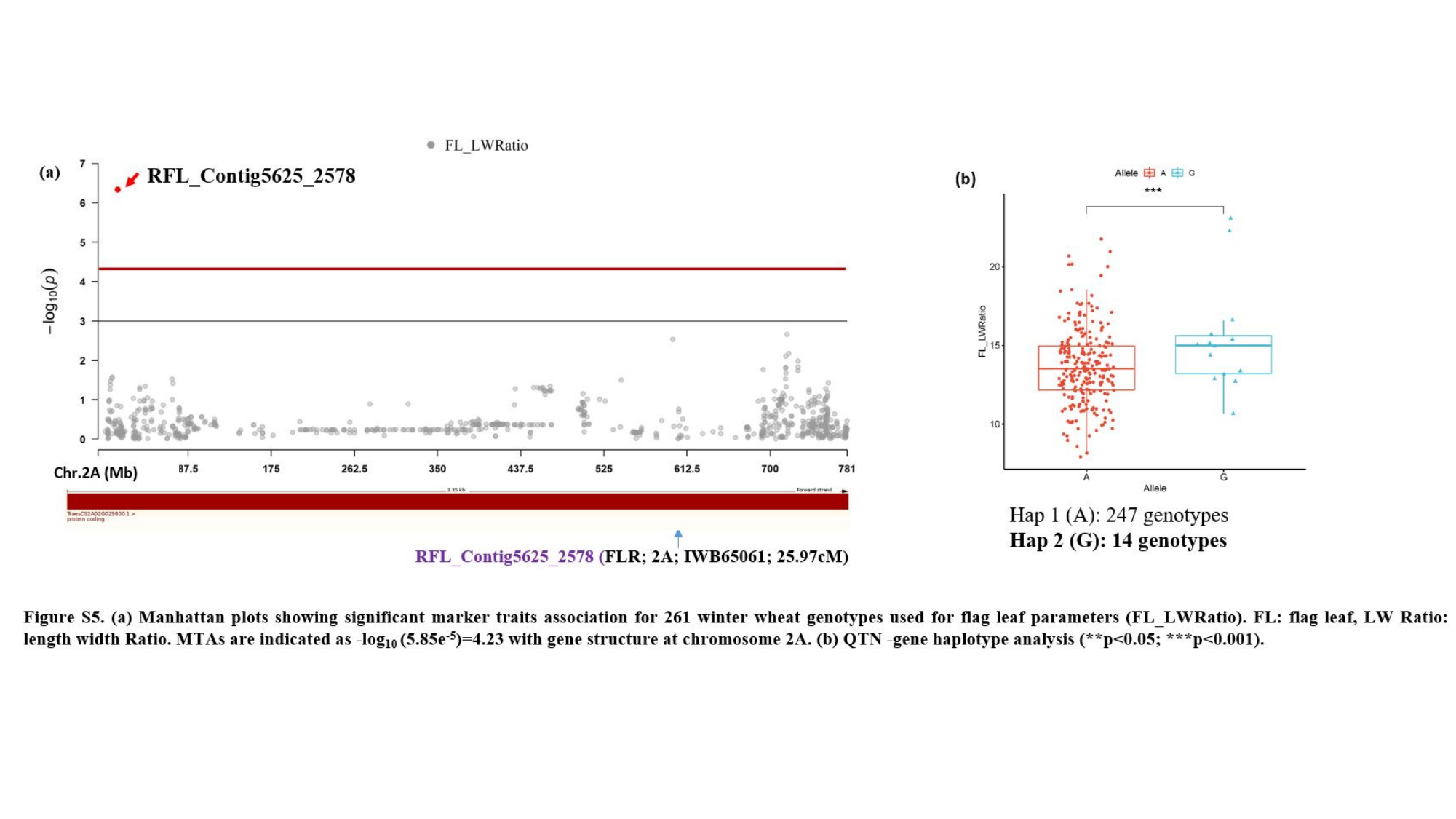

## Slide 8
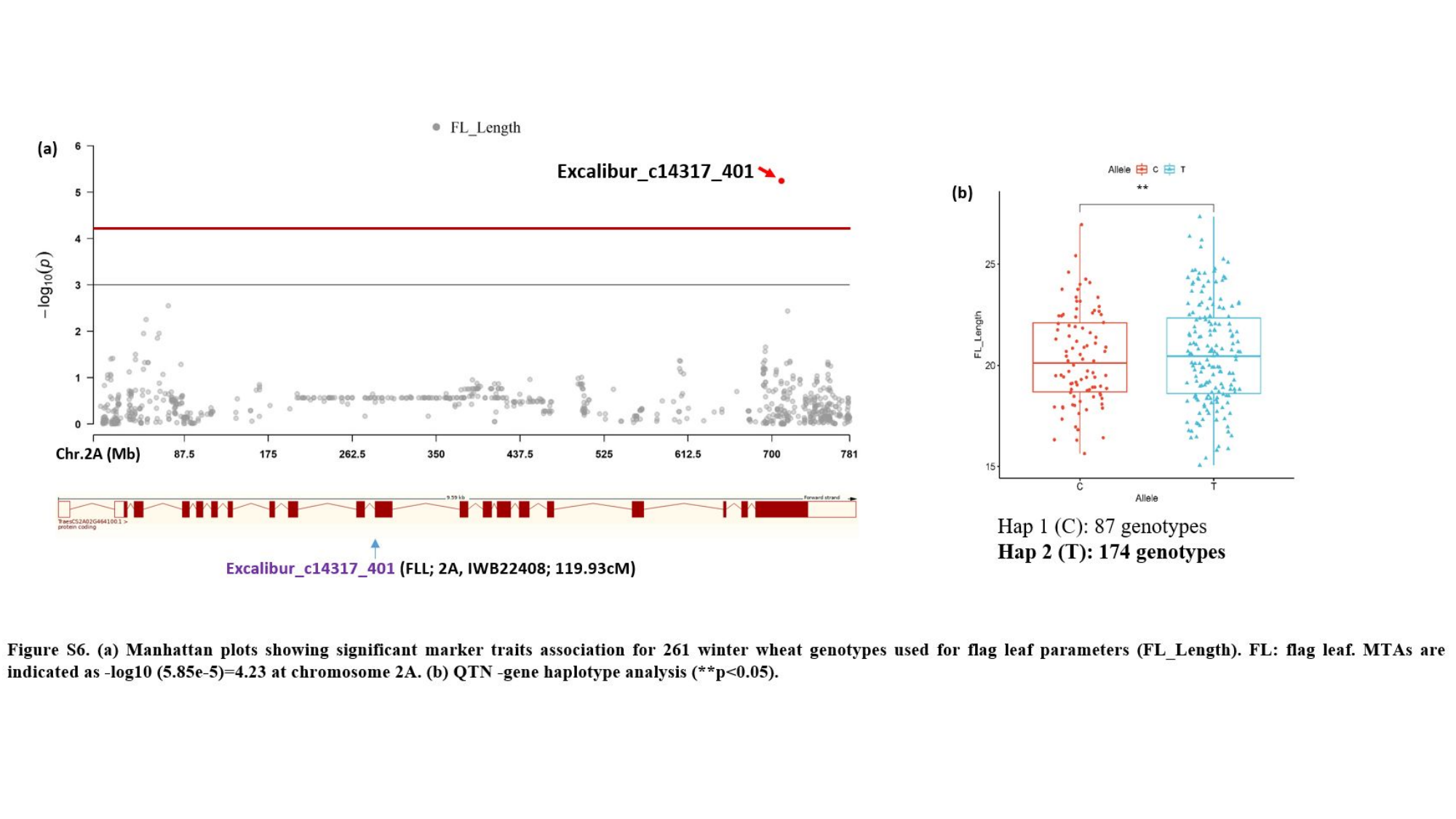

Supplement: Supplementary file 1 — Supplementary Figures. [file 41598_2024_64161_MOESM1_ESM.pptx]
