# Supplementary material for: Natural allelic variation confers diversity in the regulation of flag leaf traits in wheat
Source: Sci Rep. 2024 Jun 10;14:13316. doi: 10.1038/s41598-024-64161-x (PMC11164900; doi:10.1038/s41598-024-64161-x)
Supplement: Supplementary file 2 — Supplementary Table 1. [file 41598_2024_64161_MOESM2_ESM.docx]

***Natural allelic variation confers diversity in the regulation of flag leaf traits in wheat***

**Matías Schierenbeck^1,2,3^*, Ahmad M. Alqudah^4^*****,** **Samar G. Thabet^5^, Evangelina G. Avogadro^1^, Juan Ignacio Dietz^3,6^, María Rosa Simón^2,3^ and Andreas Börner^1^**

^1^ Leibniz Institute of Plant Genetics and Crop Plant Research (IPK), OT Gatersleben, Corrensstr 3, D-06466 Seeland, Germany.

^2^ Faculty of Agricultural Sciences and Forestry, National University of La Plata, La Plata, Argentina.

^3^ CONICET CCT La Plata. La Plata, Buenos Aires, Argentina.

^4^Biological Science Program, Department of Biological and Environmental Sciences, College of Art and Science, Qatar University, Doha, Qatar

^5^Department of Botany, Faculty of Science, Fayoum University, Egypt

^6^EEA INTA Bordenave. Ruta 76 km 36. Bordenave, Buenos Aires, Argentina

*corresponding author email:

**Matías Schierenbeck**: [m_schierenbeck@hotmail.com](mailto:m_schierenbeck@hotmail.com); schierenbeck@ipk-gatersleben.de

**Ahmad M. Alqudah^:^** [aalqudah@qu.edu.qa](mailto:aalqudah@qu.edu.qa); [ahqudah@gmail.com](mailto:ahqudah@gmail.com)

**Table S1. Winter wheat association panel Information (source, country of origin and biological status of the accessions)**

| CODE | Genotype name | Source | Country code and Region | Biological Status |
| --- | --- | --- | --- | --- |
| FW1 | Cook | CoreCollection NoviSad via IPK | AUS (other) | cultivar |
| FW2 | Min. Dwarf | CoreCollection NoviSad via IPK | AUS (other) | cultivar |
| FW3 | Triple Dirk B (GK 12) | CoreCollection NoviSad via IPK | AUS (other) | line |
| FW4 | Triple Dirk B (GK 775) | CoreCollection NoviSad via IPK | AUS (other) | line |
| FW5 | Wagrein | n/a | AUT (CNE) | cultivar |
| FW6 | Suzor'e | NGB | BLR (EEWA) | cultivar |
| FW7 | Antonovka | GSA | BUL (EEWA) | cultivar |
| FW8 | KATYA | HLWWC | BUL (EEWA) | cultivar |
| FW9 | Kristall | GSA | BUL (EEWA) | cultivar |
| FW10 | Neda | GSA | BUL (EEWA) | cultivar |
| FW11 | Rusalka | CoreCollection NoviSad via IPK | BUL (EEWA) | cultivar |
| FW12 | Svilena | GSA | BUL (EEWA) | cultivar |
| FW13 | Zlatica | GSA | BUL (EEWA) | cultivar |
| FW14 | CDC BUTEO | HLWWC | CAN (NAm) | cultivar |
| FW15 | CDC FALCON | HLWWC | CAN (NAm) | cultivar |
| FW16 | DH01-25-135*R | HLWWC | CAN (NAm) | DH line |
| FW17 | DH01-25-199*R | HLWWC | CAN (NAm) | DH line |
| FW18 | DH01-29-167 | HLWWC | CAN (NAm) | DH line |
| FW19 | DH01-29-33*R | HLWWC | CAN (NAm) | DH line |
| FW20 | DH01-32-13 | HLWWC | CAN (NAm) | DH line |
| FW21 | DH02-15-54 | HLWWC | CAN (NAm) | DH line |
| FW22 | DH99-39-55-5* | HLWWC | CAN (NAm) | DH line |
| FW23 | DH02-18-88 | HLWWC | CAN (NAm) | DH line |
| FW24 | DH01-29-125 | HLWWC | CAN (NAm) | DH line |
| FW25 | DH99-55-342-4 | HLWWC | CAN (NAm) | DH line |
| FW26 | PEREGRINE | HLWWC | CAN (NAm) | line |
| FW27 | S01-249-14*R | HLWWC | CAN (NAm) | line |
| FW28 | S01-249-8*R | HLWWC | CAN (NAm) | line |
| FW29 | S01-285-20*R | HLWWC | CAN (NAm) | line |
| FW30 | S01-285-7*R | HLWWC | CAN (NAm) | line |
| FW31 | S01-31-12 | HLWWC | CAN (NAm) | line |
| FW32 | S01-350-6 | HLWWC | CAN (NAm) | line |
| FW33 | S01-360-1 | HLWWC | CAN (NAm) | line |
| FW34 | Lambriego Inia | CoreCollection NoviSad via IPK | CHL (other) | cultivar |
| FW35 | Peking 11 | CoreCollection NoviSad via IPK | CHN (other) | cultivar |
| FW36 | Sakura | GSA | CZ (CNE) | cultivar |
| FW37 | Simila | GSA | CZ (CNE) | cultivar |
| FW38 | AURA | NGB | FIN (CNE) | cultivar |
| FW39 | JYVÄ | NGB | FIN (CNE) | cultivar |
| FW40 | LINNA | NGB | FIN (CNE) | cultivar |
| FW41 | Vakka | NGB | FIN (CNE) | cultivar |
| FW42 | APACHE | GSA | FRA (CNE) | cultivar |
| FW43 | ARLEQUIN | GSA | FRA (CNE) | cultivar |
| FW44 | AURELE | GSA | FRA (CNE) | cultivar |
| FW45 | BUENNO | GSA | FRA (CNE) | cultivar |
| FW46 | Capelle Desprez | CoreCollection NoviSad via IPK | FRA (CNE) | cultivar |
| FW47 | Durin | CoreCollection NoviSad via IPK | FRA (CNE) | cultivar |
| FW48 | GSA1 | GSA | FRA (CNE) | line |
| FW49 | GSA10 | GSA | FRA (CNE) | line |
| FW50 | GSA3 | GSA | FRA (CNE) | line |
| FW51 | GSA5 | GSA | FRA (CNE) | line |
| FW52 | GSA7 | GSA | FRA (CNE) | line |
| FW53 | MH.98-16 | GSA | FRA (CNE) | line |
| FW54 | PICARD | GSA | FRA (CNE) | cultivar |
| FW55 | Avalon | CoreCollection NoviSad via IPK | GBR (CNE) | cultivar |
| FW56 | Brigant | CoreCollection NoviSad via IPK | GBR (CNE) | cultivar |
| FW57 | TJB 990-15 | CoreCollection NoviSad via IPK | GBR (CNE) | line |
| FW58 | AKTEUR | GSA | GER (CNE) | cultivar |
| FW59 | ARKTIS | GSA | GER (CNE) | cultivar |
| FW60 | ATHLON | GSA | GER (CNE) | cultivar |
| FW61 | BRILLIANT | GSA | GER (CNE) | cultivar |
| FW62 | DISCUS | GSA | GER (CNE) | cultivar |
| FW63 | FAMULUS | GSA | GER (CNE) | cultivar |
| FW64 | Flair | GSA | GER (CNE) | cultivar |
| FW65 | GSA12 | GSA Kursk 2009/10 | GER (CNE) | line |
| FW66 | JULIUS | GSA | GER (CNE) | cultivar |
| FW67 | LEIFFER | GSA | GER (CNE) | cultivar |
| FW68 | MAGISTER | GSA | GER (CNE) | cultivar |
| FW69 | MATRIX | GSA | GER (CNE) | cultivar |
| FW70 | Mikon | GSA | GER (CNE) | cultivar |
| FW71 | Mulan | GSA | GER (CNE) | cultivar |
| FW72 | PIKO | GSA | GER (CNE) | cultivar |
| FW73 | Sailor | GSA | GER (CNE) | cultivar |
| FW74 | SKAGEN | GSA | GER (CNE) | cultivar |
| FW75 | SW MAXI | GSA | GER (CNE) | cultivar |
| FW76 | SW TATAROS | GSA | GER (CNE) | cultivar |
| FW77 | TARSO | GSA | GER (CNE) | cultivar |
| FW78 | TIGER | GSA | GER (CNE) | cultivar |
| FW79 | TRANSIT | GSA | GER (CNE) | cultivar |
| FW80 | Tulsa | GSA | GER (CNE) | cultivar |
| FW81 | TÜRKIS | GSA | GER (CNE) | cultivar |
| FW82 | Zentos | GSA | GER (CNE) | cultivar |
| FW83 | ZOBEL | GSA | GER (CNE) | cultivar |
| FW84 | Ana | CoreCollection NoviSad via IPK | HRV (CNE) | cultivar |
| FW85 | ZG 1011 | CoreCollection NoviSad via IPK | HRV (CNE) | line |
| FW86 | ZG K 3/82 | CoreCollection NoviSad via IPK | HRV (CNE) | line |
| FW87 | ZG K 238/82 | CoreCollection NoviSad via IPK | HRV (CNE) | line |
| FW88 | ZG K T 159/82 | CoreCollection NoviSad via IPK | HRV (CNE) | line |
| FW89 | Bankut 1205 | CoreCollection NoviSad via IPK | HUN (CNE) | cultivar |
| FW90 | L - 1 | CoreCollection NoviSad via IPK | HUN (CNE) | line |
| FW91 | Szegedi 768 | CoreCollection NoviSad via IPK | HUN (CNE) | cultivar |
| FW92 | Suwon 92 | CoreCollection NoviSad via IPK | IND (other) | cultivar |
| FW93 | Acciaio | CoreCollection NoviSad via IPK | ITA (CNE) | cultivar |
| FW94 | Ai-bian | CoreCollection NoviSad via IPK | JPN (other) | cultivar |
| FW95 | Norin 10 | CoreCollection NoviSad via IPK | JPN (other) | cultivar |
| FW96 | Karabalikskaya Osimaya | GSA | KAZ (EEWA) | cultivar |
| FW97 | KARABALYKSKAYA 101 | HLWWC | KAZ (EEWA) | cultivar |
| FW98 | KARABALYKSKAYA OSTISTAYA | HLWWC | KAZ (EEWA) | cultivar |
| FW99 | KOMSOMOLSKAYA 103 | HLWWC | KAZ (EEWA) | cultivar |
| FW100 | Komsomolskaya 75 | GSA | KAZ (EEWA) | cultivar |
| FW101 | LUTESCENS410H39 | HLWWC | KAZ (EEWA) | cultivar |
| FW102 | LUTESCENS410H48 | HLWWC | KAZ (EEWA) | cultivar |
| FW103 | LUTESCENS410H53 | HLWWC | KAZ (EEWA) | cultivar |
| FW104 | FI-400 | NGB | KG (EEWA) | cultivar |
| FW105 | Pergaja | NGB | LTV (EEWA) | cultivar |
| FW106 | BCD 1302/83 | CoreCollection NoviSad via IPK | MDA (EEWA) | line |
| FW107 | Cajeme 71 | CoreCollection NoviSad via IPK | MEX (NAm) | cultivar |
| FW108 | SERI82 | HLWWC | MEX (NAm) | cultivar |
| FW109 | RIDA | NGB | NOR (CNE) | cultivar |
| FW110 | Almari | GSA | PL (CNE) | cultivar |
| FW111 | Batuta | GSA | PL (CNE) | cultivar |
| FW112 | Bogatka | GSA | PL (CNE) | cultivar |
| FW113 | Finezja | GSA | PL (CNE) | cultivar |
| FW114 | Kobra plus | GSA | PL (CNE) | cultivar |
| FW115 | Kohelia | GSA | PL (CNE) | cultivar |
| FW116 | Korweta | GSA | PL (CNE) | cultivar |
| FW117 | Legenda | GSA | PL (CNE) | cultivar |
| FW118 | Muszelka | GSA | PL (CNE) | cultivar |
| FW119 | Muza | GSA | PL (CNE) | cultivar |
| FW120 | Naridana | GSA | PL (CNE) | cultivar |
| FW121 | Narobna | GSA | PL (CNE) | cultivar |
| FW122 | Roma | GSA | PL (CNE) | cultivar |
| FW123 | Rywalka | GSA | PL (CNE) | cultivar |
| FW124 | Slawa | GSA | PL (CNE) | cultivar |
| FW125 | Smuga | GSA | PL (CNE) | cultivar |
| FW126 | Sukces | GSA | PL (CNE) | cultivar |
| FW127 | Tonacja | GSA | PL (CNE) | cultivar |
| FW128 | Turnia | GSA | PL (CNE) | cultivar |
| FW129 | Wydma | GSA | PL (CNE) | cultivar |
| FW130 | Crina | GSA | ROM (CNE) | cultivar |
| FW131 | 7017 | IC&G SB RAS | RUS (EEWA) | line |
| FW132 | 7109 | IC&G SB RAS | RUS (EEWA) | line |
| FW133 | 7114 | IC&G SB RAS | RUS (EEWA) | line |
| FW134 | 88-85 | HLWWC | RUS (EEWA) | line |
| FW135 | 88-86 | HLWWC | RUS (EEWA) | line |
| FW136 | Al'bina 45 | HLWWC | RUS (EEWA) | cultivar |
| FW137 | Bagrationovskaya | IC&G SB RAS | RUS (EEWA) | cultivar |
| FW138 | Bashkirskaya 10 | VIR | RUS (EEWA) | cultivar |
| FW139 | Bezenchukskaja 380 | GSA | RUS (EEWA) | cultivar |
| FW140 | Bezenchukskaya 380 | VIR | RUS (EEWA) | cultivar |
| FW141 | Bezenchukskaya 616 | VIR | RUS (EEWA) | cultivar |
| FW142 | Bezenchukskaya 790 | VIR | RUS (EEWA) | cultivar |
| FW143 | Bezostaja 1 | CoreCollection NoviSad via IPK | RUS (EEWA) | cultivar |
| FW144 | BEZOSTAYA | HLWWC | RUS (EEWA) | cultivar |
| FW145 | Bezostaya 1 | VIR | RUS (EEWA) | cultivar |
| FW146 | Biruza | GSA | RUS (EEWA) | cultivar |
| FW147 | Biryuza | VIR | RUS (EEWA) | cultivar |
| FW148 | BULAVA | HLWWC | RUS (EEWA) | cultivar |
| FW149 | Doneko | GSA | RUS (EEWA) | cultivar |
| FW150 | Donska polupat. | CoreCollection NoviSad via IPK | RUS (EEWA) | cultivar |
| FW151 | Dzhangal | VIR | RUS (EEWA) | cultivar |
| FW152 | Ermak | GSA | RUS (EEWA) | cultivar |
| FW153 | Ershovskaya 11 | VIR | RUS (EEWA) | cultivar |
| FW154 | Favoritka | GSA | RUS (EEWA) | cultivar |
| FW155 | Filatovka | IC&G SB RAS | RUS (EEWA) | cultivar |
| FW156 | Guberniya | VIR | RUS (EEWA) | cultivar |
| FW157 | Irkutskaya ozimaya | IC&G SB RAS | RUS (EEWA) | cultivar |
| FW158 | Kalach 60 | VIR | RUS (EEWA) | cultivar |
| FW159 | Kazanskaya 285 | VIR | RUS (EEWA) | cultivar |
| FW160 | Kazanskaya 560 | VIR | RUS (EEWA) | cultivar |
| FW161 | Kirgiskaja 16 | GSA | RUS (EEWA) | cultivar |
| FW162 | KP134-3 | HLWWC | RUS (EEWA) | line |
| FW163 | Kuibyshevka | VIR | RUS (EEWA) | cultivar |
| FW164 | Kulundinka | IC&G SB RAS | RUS (EEWA) | cultivar |
| FW165 | Levoberezhnaya 1 | VIR | RUS (EEWA) | cultivar |
| FW166 | Levoberezhnaya 3 | VIR | RUS (EEWA) | cultivar |
| FW167 | M-31*/Jo3088 | IC&G SB RAS | RUS (EEWA) | line |
| FW168 | M-31/Cloud | IC&G SB RAS | RUS (EEWA) | line |
| FW169 | M-31/Flex | IC&G SB RAS | RUS (EEWA) | line |
| FW170 | M-31/Fox | IC&G SB RAS | RUS (EEWA) | line |
| FW171 | M-31/Holley | IC&G SB RAS | RUS (EEWA) | line |
| FW172 | Malakhit | VIR | RUS (EEWA) | cultivar |
| FW173 | Malahit | GSA | RUS (EEWA) | cultivar |
| FW174 | Moskovskaja 56 | GSA | RUS (EEWA) | cultivar |
| FW175 | Moskovskaya 40 | VIR | RUS (EEWA) | cultivar |
| FW176 | Moskovskaya 56 | VIR | RUS (EEWA) | cultivar |
| FW177 | Nemchinovskaya 24 | VIR | RUS (EEWA) | cultivar |
| FW178 | Nemchinovskaya 57 | VIR | RUS (EEWA) | cultivar |
| FW179 | Novosibirskaya 32 | IC&G SB RAS | RUS (EEWA) | cultivar |
| FW180 | Povolzhskaya 86 | VIR | RUS (EEWA) | cultivar |
| FW181 | Rannyaya 12 | VIR | RUS (EEWA) | cultivar |
| FW182 | Resurs | VIR | RUS (EEWA) | cultivar |
| FW183 | Santa | VIR | RUS (EEWA) | cultivar |
| FW184 | Saratovskaya 90 | VIR | RUS (EEWA) | cultivar |
| FW185 | Saratovskaya 17 | VIR | RUS (EEWA) | cultivar |
| FW186 | Severodonetskaya Yubileinaya | VIR | RUS (EEWA) | cultivar |
| FW187 | Skipetr | GSA | RUS (EEWA) | cultivar |
| FW188 | Skorospekla 35 | VIR | RUS (EEWA) | cultivar |
| FW189 | Smuglyanka | VIR | RUS (EEWA) | cultivar |
| FW190 | Svetoch | VIR | RUS (EEWA) | cultivar |
| FW191 | Ul'yanovka | IC&G SB RAS | RUS (EEWA) | cultivar |
| FW192 | UMKA | HLWWC | RUS (EEWA) | cultivar |
| FW193 | Viktoriya 95 | VIR | RUS (EEWA) | cultivar |
| FW194 | Volzhskaya K | VIR | RUS (EEWA) | cultivar |
| FW195 | ZAURALSKAYA OZIMAYA | HLWWC | RUS (EEWA) | cultivar |
| FW196 | Zemka | VIR | RUS (EEWA) | cultivar |
| FW197 | Zvonniza | GSA | RUS (EEWA) | cultivar |
| FW198 | Ivanka | CoreCollection NoviSad via IPK | SRB (CNE) | cultivar |
| FW199 | L 1/91 | CoreCollection NoviSad via IPK | SRB (CNE) | line |
| FW200 | Mina | CoreCollection NoviSad via IPK | SRB (CNE) | cultivar |
| FW201 | Nizija | CoreCollection NoviSad via IPK | SRB (CNE) | cultivar |
| FW202 | Nov.Crvena | CoreCollection NoviSad via IPK | SRB (CNE) | cultivar |
| FW203 | Nova banatka | CoreCollection NoviSad via IPK | SRB (CNE) | cultivar |
| FW204 | NS 22/92 | CoreCollection NoviSad via IPK | SRB (CNE) | line |
| FW205 | NS 46/90 | CoreCollection NoviSad via IPK | SRB (CNE) | line |
| FW206 | NS 55-25 | CoreCollection NoviSad via IPK | SRB (CNE) | line |
| FW207 | NS 602 | CoreCollection NoviSad via IPK | SRB (CNE) | line |
| FW208 | NS 63-24 | CoreCollection NoviSad via IPK | SRB (CNE) | line |
| FW209 | NS 33/90 | CoreCollection NoviSad via IPK | SRB (CNE) | line |
| FW210 | NS 66/92 | CoreCollection NoviSad via IPK | SRB (CNE) | line |
| FW211 | NS 79/90 | CoreCollection NoviSad via IPK | SRB (CNE) | line |
| FW212 | PKB Krupna | CoreCollection NoviSad via IPK | SRB (CNE) | cultivar |
| FW213 | Pobeda | CoreCollection NoviSad via IPK | SRB (CNE) | cultivar |
| FW214 | Renesansa | CoreCollection NoviSad via IPK | SRB (CNE) | cultivar |
| FW215 | Sava | CoreCollection NoviSad via IPK | SRB (CNE) | cultivar |
| FW216 | Slavija | CoreCollection NoviSad via IPK | SRB (CNE) | cultivar |
| FW217 | Sofija | CoreCollection NoviSad via IPK | SRB (CNE) | cultivar |
| FW218 | Äring II | NGB | SWE (CNE) | cultivar |
| FW219 | BANCO | NGB | SWE (CNE) | cultivar |
| FW220 | BORG | NGB | SWE (CNE) | cultivar |
| FW221 | FOLKE | NGB | SWE (CNE) | cultivar |
| FW222 | HOLME | NGB | SWE (CNE) | cultivar |
| FW223 | KOSACK | NGB | SWE (CNE) | cultivar |
| FW224 | RENODLAT SAMMETSVETE | NGB | SWE (CNE) | cultivar |
| FW225 | SAXO | NGB | SWE (CNE) | cultivar |
| FW226 | STARKE I | NGB | SWE (CNE) | cultivar |
| FW227 | WALDE | NGB | SWE (CNE) | cultivar |
| FW228 | Kirija | GSA | UKR (EEWA) | cultivar |
| FW229 | KRUZHINKA/MV IRMA | HLWWC | UKR (EEWA) | cultivar |
| FW230 | Liona | GSA | UKR (EEWA) | cultivar |
| FW231 | Mironovska 808 | CoreCollection NoviSad via IPK | UKR (EEWA) | cultivar |
| FW232 | Mironovskaya 808 | HLWWC | UKR (EEWA) | cultivar |
| FW233 | Odesska 267 | GSA | UKR (EEWA) | cultivar |
| FW234 | Podoljanka | GSA | UKR (EEWA) | cultivar |
| FW235 | Polisska 90 | GSA | UKR (EEWA) | cultivar |
| FW236 | Powaga | GSA | UKR (EEWA) | cultivar |
| FW237 | ST.ERYHTR 1334-07 | HLWWC | UKR (EEWA) | line |
| FW238 | ZOLOTAVA/DAR ZERNOGRADA | HLWWC | UKR (EEWA) | cultivar |
| FW239 | Benni multifloret | CoreCollection NoviSad via IPK | USA (NAm) | cultivar |
| FW240 | BULK02R2B | HLWWC | USA (NAm) | line |
| FW241 | Centurk | CoreCollection NoviSad via IPK | USA (NAm) | cultivar |
| FW242 | Cheyenne | NGB | USA (NAm) | cultivar |
| FW243 | Ernie | GSA | USA (NAm) | cultivar |
| FW244 | Florida | CoreCollection NoviSad via IPK | USA (NAm) | cultivar |
| FW245 | Goldfield | GSA | USA (NAm) | cultivar |
| FW246 | Hazen | GSA | USA (NAm) | cultivar |
| FW247 | Helios | CoreCollection NoviSad via IPK | USA (NAm) | cultivar |
| FW248 | Holly E | CoreCollection NoviSad via IPK | USA (NAm) | cultivar |
| FW249 | NI98414 | HLWWC | USA (NAm) | line |
| FW250 | Norstar | GSA | USA (NAm) | cultivar |
| FW251 | Purd.5392 | CoreCollection NoviSad via IPK | USA (NAm) | line |
| FW252 | Purd.39120 | CoreCollection NoviSad via IPK | USA (NAm) | line |
| FW253 | Red Coat | CoreCollection NoviSad via IPK | USA (NAm) | cultivar |
| FW254 | Ridit | NGB | USA (NAm) | cultivar |
| FW255 | Roughrider | GSA | USA (NAm) | cultivar |
| FW256 | SD98444/SD97060 | HLWWC | USA (NAm) | line |
| FW257 | Vel | CoreCollection NoviSad via IPK | USA (NAm) | line |
| FW258 | Wesley/SD97049 | HLWWC | USA (NAm) | line |
| FW259 | n.a.: not available |  |  |  |

**Region of origin: CNE (Central & North Europe); EEWA (Eastern Europe & Western Asia); NAm (North America); Other**
